# Advances in Bioprinting to Model Immune‐Mediated Skin Diseases

**DOI:** 10.1002/adhm.202503806

**Published:** 2025-11-27

**Authors:** Andrea Ulloa‐Fernández, Marica Markovic, Julia Fernández‐Pérez, Georg Stary, Aleksandr Ovsianikov

**Affiliations:** ^1^ Research Group 3D Printing and Biofabrication Institute of Materials Science and Technology TU Wien Vienna 1060 Austria; ^2^ Austrian Cluster for Tissue Regeneration Austria; ^3^ Department of Dermatology Medical University of Vienna Vienna 1090 Austria; ^4^ CeMM Research Center for Molecular Medicine Austrian Academy of Sciences Vienna 1090 Austria; ^5^ Christian Doppler Laboratory for Chronic Inflammatory Skin Diseases Vienna 1090 Austria

**Keywords:** 3D bioprinting, immunocompetent tissue models, in vitro tissue models, inflammatory skin diseases

## Abstract

Chronic, non‐communicable inflammatory skin diseases, are a group of inflammatory conditions with high prevalence across the world and represent a significant challenge in medicine, due to the high number of patients arriving for medical consultations. The development of engineered skin tissue models aims to create advanced in vitro models that accurately recapitulate various skin disorders. Furthermore, the introduction of the 3Rs framework to reduce animal testing, alongside specific legislation intended to minimize such testing, has driven research toward developing in vitro models that closely imitate human conditions and skin pathologies, while also being suitable for testing new therapeutic and cosmetic products. In this context, advancements in bioprinting technologies, which promise to ensure consistent quality, reduce technical variances, and enable upscaling, can improve the reproducibility and performance of such models. This work discusses current advancements in the field of bioprinting of in vitro skin models with a particular focus on their application in the research of immune‐mediated diseases.

## Graphical abstract

This review explores how 3D bioprinting drives innovation in developing in vitro skin models that mimic immune‐mediated diseases. It highlights current technologies, key applications in studying skin pathologies, and emerging challenges. The review points toward future opportunities for improving disease modeling and advancing therapeutic and cosmetic testing.

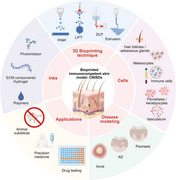

## 1 Introduction

Chronic, non‐communicable inflammatory skin diseases (CNISDs)^[^ ^1^ ^]^ constitute the largest group of chronic skin conditions influenced by genetics and the environment.^[^ ^2^, ^3^ ^]^ Approximately 25% of the European population experiences some form of inflammatory skin disorder.^[^ ^1^, ^4^ ^]^ However, despite their prevalence, the complex immune responses involved in these various disorders remain unclear. The development of in vitro skin models offers a promising alternative to animal models for research applications, including drug testing, cosmetic evaluation, and medical device assessment.^[^ ^3^ ^]^ In recent years, 3D bioprinting technologies have emerged as a groundbreaking method for creating human tissues and organs; this approach also provides physiological and architectural representations of human skin.^[^ ^5^ ^]^ Nevertheless, modeling immune responses continues to pose a challenge in enhancing the biological relevance of these models.^[^ ^3^ ^]^ This work shows an overview of the latest innovations within the 3D bioprinting in vitro skin models and underlies the need of applying immune components to in vitro models and comprehend skin inflammatory immune responses involved in several disorders. We first describe the anatomy and immune components present in the skin with their respective function of the skin along with the main inflammatory skin pathologies. We then present current models developed to study such pathologies in vitro. An overview of the main 3D bioprinting technologies used for the biofabrication of in vitro skin models is given. Finally, we discuss current challenges of the field, together with potential ways where 3D bioprinting can further contribute to the development of more physiologically relevant models.

## 2 Anatomy and Immune Function of the Human Skin

**Figure 1:**
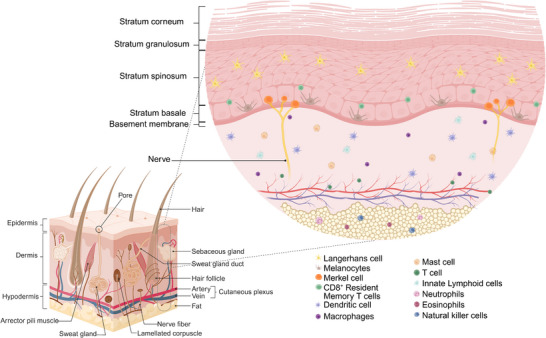
Anatomy of the skin. The skin contains three main layers: epidermis, dermis, and hypodermis. The epidermis (outermost layer) of the skin is constituted of keratinocytes layered in four strata (in palms and soles, there is an additional layer, stratum lucidum), Langerhans cells, melanocytes, and Merkel cells. The dermis is composed of fibroblasts, various subsets of lymphatic and myeloid immune cells, nerves, and vasculature, and contains appendages like sebaceous glands, sweat glands, and hair follicles. The hypodermis is composed of fat tissue and cells of the adaptive and innate immune systems. Created in BioRender. Ulloa, A. (2025) https://BioRender.com/wg6n04a.

The skin is the body's largest organ by surface area, constituting ≈16% of total body weight, and has multiple embryological origins.^[^ ^6^, ^7^ ^]^ It serves as a vital barrier that protects us from various insults such as extreme temperatures, UV rays, allergens, microbes, and toxins.^[^ ^7^ ^]^ Anatomically, the skin is structured into three layers: epidermis, dermis, and hypodermis, from the outermost to the innermost, to perform distinct functions^[^ ^8^ ^]^ and maintain the mechanical and immunological barriers, as illustrated in **Figure** **1**. Lined between the epidermis and the dermis is the basement membrane, composed of connective tissue that compartmentalizes yet connects the epidermal keratin intermediate filaments with the dermal collagen network,^[^ ^9^ ^]^. The base membrane primarily consisting of laminins, collagen IV, and VII.^[^ ^8^, ^10^, ^11^ ^]^ As the principal physical barrier, each layer contains a specific composition of immune cells contributing to the tissue homeostasis and, in the event of a danger signal, to immune responses.^[^ ^12^ ^]^ In addition, there is also an interaction with the skin microbiome, which constantly engages with keratinocytes and immune cells in the epidermis, enhancing host defense against pathogens, controlling inflammation, and stimulating adaptive immune pathways.^[^ ^13^ ^]^

### 2.1 Epidermis

The epidermis is a stratified squamous epithelium, originates from the ectodermal layer of the embryo (Figure 1). The epidermis is mainly composed of keratinocytes organized into four strata, except for palms and soles that contain an additional layer, the stratum lucidum. Each stratum is organized based on the differentiation state of keratinocytes, ranging from those that are terminally differentiated to those with proliferative capacity: stratum corneum, stratum lucidum, stratum granulosum, stratum spinosum, and stratum basale.^[^ ^7^ ^]^ As well as keratinocytes, the stratum basale contains melanocytes, which originate from the neural crest, and provide skin pigmentation and photoprotection, and Merkel cells, also called tactile epithelial cells, that are involved in tactile sensitivity. From an immunological perspective, the epidermis contains Langerhans cells, a type of antigen‐presenting cell, and primarily CD8^+^ resident memory T cells (T_RM_), specialized T cells that persist after an infection or inflammation, both in the stratum spinosum and the melanocytes in the stratum basale that play a role in antigen presentation.^[^ ^12^ ^]^

### 2.2 Dermis

The dermis layer is abundant in fiber‐forming proteins, among them collagen I, III, and elastin; adhesive glycoproteins such as fibronectin; glycosaminoglycans (GAGs) for example hyaluronic acid; and matricellular proteins that enhance mechanical strength and flexibility, while maintaining a significant amount of water to create a moist microenvironment.^[^ ^8^ ^]^ The rearrangement of conective tissue fibers distinguishes two types of dermal layers: the papillary dermis, which has thin fibers, and the reticular dermis, characterized by thick and coarse fibers.^[^ ^11^ ^]^ The dermis is composed mainly of fibroblasts, which are responsible for synthesizing the extracellular matrix (ECM). Additionally, various immune cell populations are found in the dermis and includes dendritic cells, macrophages, T cells, innate lymphoid cells, and mast cells. Appendages such as sweat glands, hair follicles, and sebaceous glands, along with other components like nerves and blood vessels that also traverse this layer and support skin function.^[^ ^5^, ^6^, ^12^ ^]^

### 2.3 Hypodermis

The deep layer of the skin is the hypodermis, situated beneath the dermis and above the muscle. Also known as the subcutaneous layer, it is primarily composed of fatty connective tissue but also contains collagen, elastin fibers, and adipocyte lobules. This layer plays a critical role in thermal insulation, energy storage, and shock absorption. It produces hormones like leptin, which help regulate the body's weight through the hypothalamus.^[^ ^5^, ^11^ ^]^ Additionally, T cells, macrophages, neutrophils, natural killer cells, and eosinophils are present in the hypodermis, aiding in the development of wound healing.^[^ ^14^, ^15^ ^]^

The skin provides physical and immune defense while also playing a key role in thermoregulation and sensation.^[^ ^12^, ^16^ ^]^ In addition to accessory structures like sweat glands, nails, hair, and sebaceous glands, this organ is crucial for the absorption, excretion, and secretion of a variety of substances.

### 2.4 Cellular Interplay in the Cutaneous Immune System

Within the various layers of the skin, several populations of immune cells continuously interact with each other and with structural cells and produce effector cytokines and regulatory factors for the resident skin tissue cells, which are responsible for regulating, recruiting, and activating immune cells when necessary. Together, they form a network that maintains and promotes barrier homeostasis.^[^ ^17^, ^18^ ^]^

The epidermis is a dynamic structure that responds to environmental stimuli to attract or repel immune cells via keratinocytes and hair follicles, while Langerhans cells and T_RM_ continuously sense the microenvironment.^[^ ^7^, ^19^ ^]^ Keratinocytes are a crucial component of the innate immune system; they exhibit various pattern recognition receptors and produce an array of cytokines to promote recruitment and regulate the survival of immune cells through the secretion of survival factors like colony‐stimulating factor 1 (CSF‐1), as well as tumor necrosis factor (TNF), interleukin‐33 (IL‐33), and other interleukin‐1 family members (IL‐1) that contribute to immune activation and immune cell recruitment.^[^ ^7^, ^18^, ^20^, ^21^ ^]^

The vasculature in the dermal compartment features post‐capillary venules with specific permeability characteristics, allowing access to the skin exclusively for crucial mediators when humoral immunity is compromised during inflammatory conditions. The skin's nervous system, via sensory neurons, regulates the immune functions of dendritic cells. Finally, the hypodermis, made up of adipose tissue, controls hair growth, which contributes to antibacterial host defense through the production of cathelicidin.^[^ ^18^ ^]^

Related to the skin microbiome, microbial colonization begins at birth. There are variations in temperature, pH, humidity, and the density of sebaceous glands throughout the body surface, creating an ideal habitat for various microorganisms such as bacteria, fungi, viruses, archaea, and mites.^[^ ^22^ ^]^ The interaction among different commensal species on the skin can stimulate the release of keratinocyte antimicrobial peptides (AMPs) and inhibit the release of inflammatory cytokines during wound healing,^[^ ^13^ ^]^ or prevent the colonization of pathogenic or opportunistic microbes, thus modulating the immune response and maintaining skin homeostasis.^[^ ^17^, ^22^ ^]^ However, dysbiosis (an imbalance among microbial communities) can activate the immune system and lead to skin conditions like atopic dermatitis or acne.^[^ ^13^, ^18^ ^]^

## 3 Inflammatory Skin Pathologies

CNISDs are widespread in the global population and represent a major challenge in medicine. CNISDs can arise from an interaction between genetic and environmental factors, such as psoriasis, atopic dermatitis, acne, hidradenitis suppurativa, urticaria, lichen planus, rosacea, and sarcoidosis.^[^ ^1^, ^23^ ^]^ Another group of CNISDs can result from different autoimmune dysregulations, such as vitiligo or alopecia areata,^[^ ^24^, ^25^ ^]^ where the autoimmunity is mediated by T cells or B cells, as seen in chronic spontaneous urticaria and pemphigoid diseases.^[^ ^26^, ^27^ ^]^ Other conditions, such as diabetes^[^ ^28^ ^]^ or rheumatism, can also affect skin homeostasis and trigger inflammatory complications. Advancements in tissue engineering technologies, along with the incorporation of immune components, would help to provide more physiologically relevant in vitro models that better reflect the immunopathology of various skin disorders, thereby improving preclinical research and drug discovery.^[^ ^29^, ^30^ ^]^

In this work, we describe the most representative CNISDs that are currently being modeled or show promising applicability for future modeling using 3D bioprinting technologies, with a particular focus on the critical role of the immune component in the development of these models.

### 3.1 Psoriasis

Psoriasis is a chronic T‐cell‐mediated inflammatory disease that affects ≈2% of the global population, triggered by genetic and environmental factors.^[^ ^31^, ^32^, ^33^ ^]^ The mortality rate is low, and while it primarily affects the skin, epidemiological studies link psoriasis with other chronic diseases such as vascular comorbidities, nerve damage, metabolic syndrome, and psoriatic arthritis.^[^ ^31^, ^34^ ^]^ In dark‐skinned patients, the lesions tend to be thicker, cover a larger body area, and exhibit desquamation.^[^ ^35^ ^]^ Patients experience itchy, painful lesions that significantly impair their quality of life and contribute to psychosocial burden and depression.^[^ ^34^, ^36^ ^]^ Psoriatic plaques change the skin's morphology through aberrant differentiation of keratinocytes, overproliferation, retention of nuclei in the stratum corneum (parakeratosis), and thickening of the epidermis (acanthosis), resulting in the formation of elevated erythematous plaques, elongated rete ridges, and desquamation^[^ ^31^ ^]^ (**Table** **1**).

**Table 1:** Summary of main features and risk factors of selected CNISDs.

CNISD	Main features	Risk factors
Psoriasis	– Th1/Th17‐ driven inflammation – Erythematous plaques – Desquamation – Keratinocyte hyperproliferation – Parakeratosis – Acanthosis – Elongated rete ridges	– Genetic and environmental factors – Medications (psoriasis‐inducing or exacerbating drugs) – Metabolic and lifestyle‐related risk factors (obesity, smoking, alcohol consumption)
Atopic dermatitis	– Th2‐driven inflammation – Type I hypersensitivity reaction – Pruritus – Eczema – Dry skin – IgE elevated serum levels – Spongiosis – Lichenification – Keratinocyte hyperproliferation – Barrier dysfunction (↓ Filaggrin expression) – Parakeratosis – Dysbiosis (↑ *S. aureus*)	– Genetic predisposition (e.g., filaggrin mutations) – Environmental factors (allergen exposure in food allergies, asthma, rhinitis) – Skin microbiome dysbiosis
Acne	– Presence of comedones, papules, pustules, nodules, cysts – Increase in sebum production – Alteration of sebum composition – Dysbiosis (↑ *C. acnes*) – Keratinocyte hyperproliferation – Th17‐driven inflammation	– Genetic – Hyperhidrosis – Androgen‐driven hypersecretion of sebum – Premenstrual exacerbation – ↑ dairy, fatty, sugary products intake – Smoking – Stress – Medications – Unsuitable skincare for skin type – Seasonal factors – Lack of sleep
Hidradenitis suppurativa	– Lesions in skin folds – Inflamed nodules, abscesses – Malodorous – Sinus tracts and fistulas – Persistent suppuration – Hypertrophic scars	– Genetic predisposition – Immune dysregulation – Hormonal imbalances
Vitiligo	– Depigmented patches on the body – Activation of autoreactive CD8^+^ T cells against melanocytes – CD8^+^ T cells induced apoptosis	– Genetic predisposition – T cell‐mediated autoimmunity – Oxidative stress – Catalase and SOD deficiencies – Exposure to drugs and chemicals – Neurological system impairment
Chronic spontaneous urticaria	– Mast cell‐driven inflammation – Erythematous wheals – Angioedema – Itchy – Vasodilation – Histamine, leukotrienes, platelet‐activating factor, and prostaglandin D2 release – Th1/Th17/Th2 immune response	– Immune dysregulation – Chronic infections as predisposition factor

**Figure 2:**
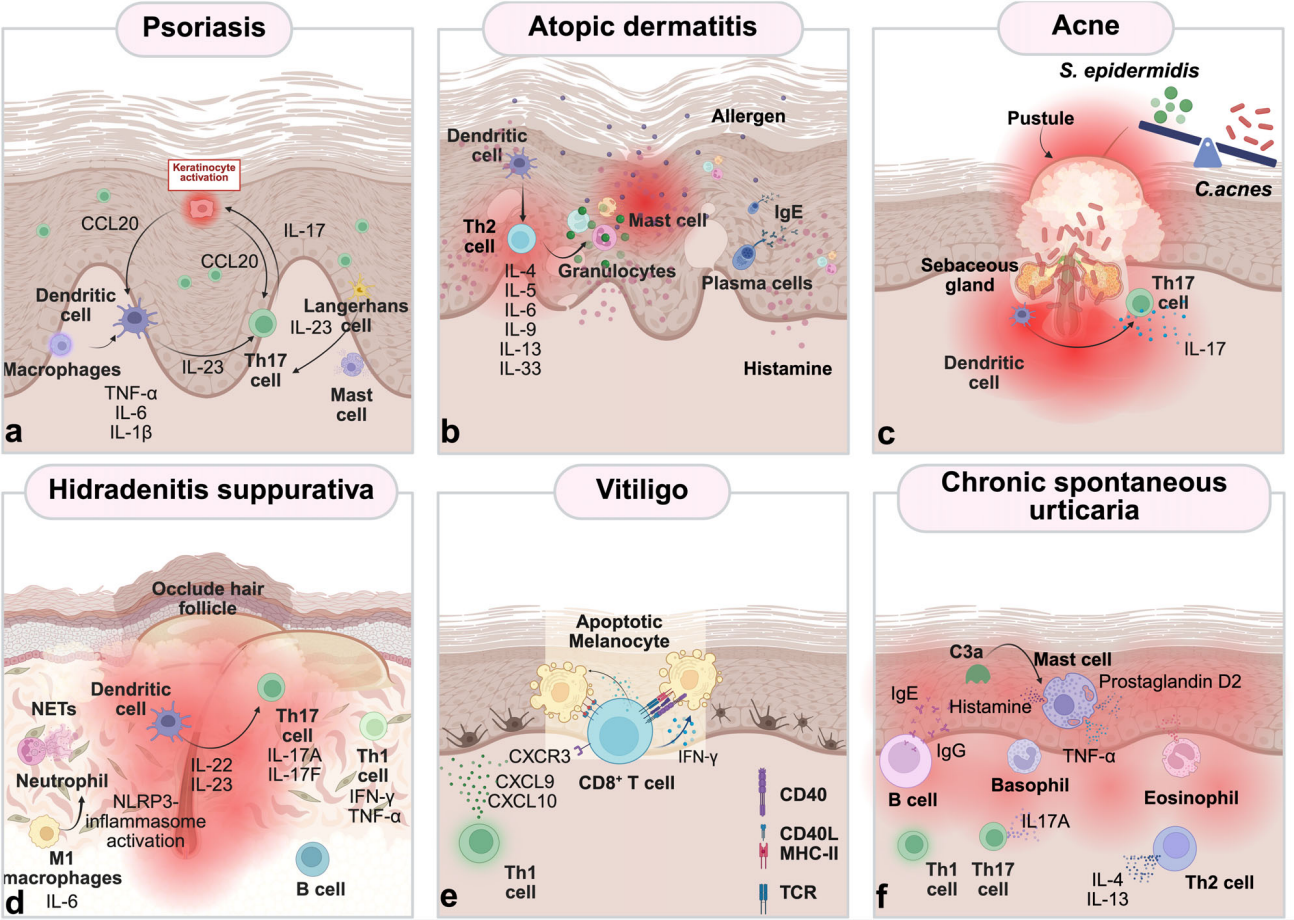
Key immunopathological processes in the main inflammatory skin disorders. Triggered by genetic and environmental factors: a) Psoriasis, a Th17‐cell‐mediated inflammatory disease; b) Atopic Dermatitis, driven by Th2 immune activation; c) Acne, driven by hormonal, microbial, and immunological factors; d) Hidradenitis suppurativa, an inflammatory condition that involves follicular occlusion and dysregulation in innate and adaptive immune reactions. Triggered by autoimmune dysregulation: e) Vitiligo, an autoimmune T‐cell mediated depigmenting skin disorder; f) Chronic spontaneous urticaria, a mast cell‐driven inflammatory disease. Created in BioRender. Ulloa, A. (2025) https://BioRender.com/w73ko38.

A crosstalk between keratinocytes and various immune cell populations has been characterized, playing a role in the initiation and maintenance of inflammation.^[^ ^36^ ^]^ As part of the innate immune response, stressed keratinocytes release AMPs that activate dendritic cells^[^ ^36^ ^]^ which then begin secreting interferon‐alpha (IFN‐α), interferon‐gamma (IFN‐γ), tumor necrosis factor‐alpha (TNF‐α), interleukin 1‐beta (IL‐1β), interleukin 12 (IL‐12), and interleukin 23 (IL‐23), activating the response of T helper cells 1 and 17 (Th1 and Th17).^[^ ^34^, ^37^ ^]^ Th1 and Th17 cells subsequently secrete TNF‐α, interleukin 17 (IL‐17), interleukin 21 (IL‐21), and interleukin 22 (IL‐22), creating a loop of keratinocyte activation and secretion of AMPs, cytokines, and chemokines like C‐C motif chemokine ligand 20 (CCL20), amplifying the proinflammatory response, T‐cell infiltration and inducing leukocyte recruitment^[^ ^36^, ^38^, ^39^ ^]^ (**Figure** **2**a).

In the dermis, there is an increase in vascular proliferation, overstimulation of the sensory nervous system, infiltration of various subsets of proinflammatory cells, including T‐cells and macrophages, and the activation of dendritic cells.^[^ ^31^ ^]^ Fibroblasts located at the tips of the dermal papillae secrete proinflammatory cytokines, and together with endothelial cells, they induce tissue remodeling and the generation of hyperplastic blood vessels that contribute to epidermal dysregulation.^[^ ^38^, ^39^ ^]^

Along with immune and vascular abnormalities, several histological biomarkers can be identified in psoriatic lesions, such as a reduction of the granular epithelium and downregulation of the cytokeratin 10 cell marker in the stratum basale. Furthermore, there is a significant upregulation of the proliferative cell marker Ki67 and cytokeratin 16, which indicates hyperproliferation and aberrant keratinocyte differentiation. An increase in the expression of AMPs, such as S100A7 (psoriasin) and elafin, is also observed in psoriatic lesions.^[^ ^33^, ^40^ ^]^

### 3.2 Atopic Dermatitis

Atopic dermatitis (AD) is a T‐cell‐mediated inflammatory disease that affects individuals at various stages of life, with a higher prevalence in children, where 15–20% develop lesions, and ≈10% of adults also present lesions. These rates are consistently increasing in urbanized and developing countries, causing mental and economic challenges for patients and their families.^[^ ^2^, ^41^ ^]^ It is one of the most common allergy‐mediated inflammatory diseases,^[^ ^33^ ^]^ manifesting from a combination of predisposing genetic, immunological, and environmental factors, as well as significant skin microbiome dysbiosis.^[^ ^42^ ^]^ Current evidence indicates a correlation between AD and food allergies, asthma, rhinitis, and comorbidities such as cardiovascular and gastrointestinal diseases.^[^ ^43^ ^]^

Patients with AD lesions experience pruritus, chronic eczema that varies in severity among individuals, dry skin, infections caused by *Staphylococcus aureus*, and elevated serum levels of Immunoglobulin E (IgE).^[^ ^41^, ^44^ ^]^ Itchy rash lesions can result in spongiosis, lichenification, keratinocyte hyperproliferation, and parakeratosis (Table 1). The pathological mechanism begins with a barrier dysfunction due to mutations in the gene responsible for filaggrin or other factors that disrupt keratinocyte terminal differentiation, allowing the penetration of cutaneous antigens, microbial products, and environmental pollutants that can activate the immune system and initiate a lesion.^[^ ^45^ ^]^

Once the immune system is activated, a typical Type I hypersensitivity reaction occurs. There is an increase in the influx of T helper‐2 cells (Th2) to the epithelium. Th2 cells begin secreting IL‐4, IL‐5, IL‐6, IL‐9, IL‐13, and IL‐33, which recruit other immune cells, such as granulocytes (neutrophils and eosinophils), and mast cells. This process causes the secretion of IgE from plasma cells, as well as the degranulation of mast cells, releasing histamine and other proinflammatory mediators^[^ ^17^, ^33^, ^44^, ^46^ ^]^ (Figure 2b).

These pathological changes in the skin microenvironment are accompanied by distinct molecular markers, including an increase in Thymic Stromal Lymphopoietin, the release of cytokines, and decreases in filaggrin expression, which impair barrier function.^[^ ^33^ ^]^

### 3.3 Acne

Acne is an inflammatory condition that is highly prevalent among teenagers but is increasingly found in adults worldwide. It is characterized by the presence of various inflammatory and non‐inflammatory lesions, including comedones, papules, pustules, nodules, and cysts.^[^ ^47^ ^]^ It typically appears in areas with a high density of sebaceous glands, such as the face, arms, and back, resulting in varying degrees of scarring and hyperpigmentation.^[^ ^48^ ^]^ The causes are multifactorial; genetic predisposition has been indicated, along with hyperhidrosis, androgen‐driven hypersecretion of sebum, premenstrual exacerbation, and lifestyle habits such as high intake of dairy products, sugary and fatty foods, smoking, stress, medications, specific cosmetics and moisturizers, seasonal factors, and lack of sleep, all of which contribute to acne progression through increased sebum production and alterations in sebum composition^[^ ^47^, ^49^, ^50^ ^]^ (Table 1).

Sebaceous glands (SG) play a critical role in the development of acne lesions, alongside dysbiosis. Sebocytes, the primary cells located in SG, regulate the activity and accumulation of sex hormones as well as the expression of androgen receptors in the skin.^[^ ^51^ ^]^ They produce sebum, an oily secretion that insulates, smooths, and protects the skin while also possessing antimicrobial and antioxidant properties.^[^ ^52^ ^]^

The pathogenesis occurs when the balance between *Cutibacterium acnes* and *Staphylococcus epidermidi*s is disrupted. S. *epidermidi*s inhibits *C. acnes* proliferation by producing succinic acid. Conversely, *C. acnes* restricts *S. epidermidi*s growth by releasing propionic acid near the pilosebaceous follicle. The loss of equilibrium in favor of the more virulent phylotypes of *C. acnes* in the skin, results in the production of proinflammatory mediators, which induce follicular keratinocyte hyperproliferation,^[^ ^22^, ^50^ ^]^ the priming of dendritic cells that drive the polarization of CD4 naïve T cells to Th17, and the subsequent secretion of IL‐17^[^ ^18^, ^53^, ^54^ ^]^ (Figure 2c).

### 3.4 Hidradenitis Suppurativa

Hidradenitis suppurativa (HS) is a chronic inflammatory disease commonly known as acne inversa. Lesions typically emerge in early adulthood and can last for decades. The global prevalence is 0.4%, with higher rates among females and African Americans.^[^ ^55^, ^56^ ^]^ These lesions are often located in skin folds, including the armpits, breasts, groin, gluteal area, and perianal region.^[^ ^57^, ^58^ ^]^ Patients suffer from severe pain, restricted movement, and a significant negative impact on their quality of life and mental health. The lesions are defined by painful, inflamed nodules, abscesses, and malodorous, pus‐filled tunnels known as sinus tracts and fistulas that extend deeply into the dermis, leading to persistent suppuration. The sinus tracts undergo multiple cycles of rupture and repair, resulting in the formation of hypertrophic scars and fibrosis^[^ ^1^, ^57^, ^58^ ^]^ (Table 1).

The pathogenesis of HS is complex, involving genetic predisposition, lifestyle factors, immune dysregulation, and microbial and hormonal imbalances that cause blockage of hair follicles.^[^ ^59^, ^60^ ^]^ The occluded follicles result in recurrent cycles of rupture and wound formation, leading to immune activation characterized by neutrophil infiltration and the formation of neutrophil extracellular traps (NETs), which increase the inflammation through the activation of the NLRP3‐inflammasome pathway.^[^ ^61^ ^]^ The inflammatory mediators released trigger the activation and recruitment of dendritic cells (IL‐23), M1 macrophages, Th1 cells (IFN‐γ and TNF‐α), Th17 cells (IL‐17A, IL‐17F, IL‐22, and IL‐6), as well as T and B cells. This leads to widespread pus production, irreversible tissue damage, and the formation of scar tissue (Figure 2d).^[^ ^57^, ^58^, ^62^ ^]^

### 3.5 Vitiligo

Vitiligo is a chronic skin disorder characterized by depigmentation that affects the melanocytes in the epidermis, impacting ≈2% of individuals across various populations.^[^ ^25^ ^]^ Vitiligo is a non‐transmissible polygenic disease with several pathophysiological factors in its progression, among them T‐cell‐mediated autoimmunity, oxidative stress, genetic predisposition, exposure to drugs and chemicals, and impairment of the neurological system. Clinically, it presents as well‐defined white patches on the body, which can lead to psychological distress.^[^ ^1^, ^63^ ^]^

The most widely accepted mechanism described in the pathophysiology of vitiligo involves the activation of autoreactive CD8^+^ T cells that target specific melanocyte antigens, resulting in cytotoxicity.^[^ ^64^ ^]^ This mechanism is mediated by IFN‐γ, which acts on melanocytes. IFN‐γ stimulates the secretion of Th1 chemokines CXCL9 and CXCL10, which bind to CXCR3 on cytotoxic CD8^+^ T cells and melanocytes, establishing a positive feedback loop that recruits additional T cells and induces apoptosis in melanocytes. Further stimulation by IFN‐γ can enhance the expression of CD40, CD80, and MHC II, leading to the presentation of self‐antigens in melanocytes, which increases T cell proliferation and drives the development of adaptive immune responses.^[^ ^25^, ^65^, ^66^ ^]^ Other theories also link increased oxidative stress to deficiencies in antioxidant enzymes such as catalase and superoxide dismutase (SOD), making melanocytes even more susceptible to apoptosis and amplifying the expression of MHC I, thereby increasing vulnerability to CD8^+^ T cell‐induced apoptosis (Figure 2e) (Table 1).^[^ ^67^ ^]^

### 3.6 Chronic Spontaneous Urticaria

Chronic spontaneous urticaria (CSU) is an inflammatory disease driven by mast cells. It is characterized by the unpredictable formation of itchy, raised, erythematous wheals and, in some cases, angioedema lasting more than six weeks, with spontaneous remission occurring after several years.^[^ ^1^ ^]^ These symptoms result from the stimulation of sensory nerves, vasodilation, and increased local vascular permeability.^[^ ^68^ ^]^ Mast cells are the primary cells involved in the pathology of CSU. The activation and degranulation of mast cells lead to the release of histamine and other vasoactive mediators, including leukotrienes, platelet‐activating factor, and prostaglandin D2 (PGD2), as well as various cytokines and chemokines that mimic a Th2 immune response, along with proteases such as chymase and tryptase^[^ ^68^, ^69^ ^]^ (Table 1).

Another characteristic of CSU is the infiltration of basophils, eosinophils, monocytes, T cells, B cells, and neutrophils, accompanied by a proinflammatory profile. This profile induces a Th1/Th17/Th2 immune response that encourages the overactivation of mast cells.^[^ ^68^ ^]^ Mast cell activation can occur through various pathways, including the production of autoantibodies, the coagulation cascade, and the complement system (C3a fragment), as well as mechanisms mediated by IgE and IgG mechanisms^[^ ^70^ ^]^ resulting from B‐cell activity. This activity subsequently increases proliferation, class switching, and autoantibody production, which also influences CSU pathogenesis (Figure 2f).^[^ ^70^, ^71^ ^]^

In addition to the CNIDs mentioned, systemic conditions also contribute to skin disorders through their pathophysiological mechanisms. Among these, diabetes mellitus plays a significant role. Chronic hyperglycemia in diabetes leads to protein glycosylation of structural proteins like collagen, and production of glycosylation end products that induce an increase in oxidative stress. The collagen glycosylated loose elasticity and became resistant to degradation and renewal, affecting the mechanical properties in the skin.^[^ ^72^, ^73^, ^74^ ^]^ Additionally, hyperglycemia is associated with impaired blood circulation, which contributes to chronic inflammation, affects wound healing, alters proliferation, keratinocyte differentiation, and the proper secretion of ECM and adipose hypertrophy.^[^ ^28^ ^]^ These effects compromise the microvasculature, promote neuropathy, and lead to immune‐mediated skin damage.^[^ ^72^, ^75^ ^]^ The immune mechanisms, systemic‐cutaneous interactions, and overlapping signaling pathways, such as those potentially shared with psoriasis^[^ ^76^ ^]^ and atopic dermatitis,^[^ ^77^ ^]^ in diabetic skin remain poorly understood.

Several animal models for different skin conditions have been created and extensively studied. However, the lack of reproducibility and the anatomical and immunological differences between species result in low translational relevance.^[^ ^78^, ^79^, ^80^ ^]^ Moreover, the implementation of the 3Rs principles (Directive 2003/15/EC of the European Parliament and of the Council of 27 February 2003 amending Council Directive 76/768/EEC on the approximation of the laws of the Member States relating to cosmetic products^[^ ^81^ ^]^; European Regulation 1223/2009^[^ ^82^ ^]^; The United States Federal Food, Drug, and Cosmetic Act, 2022^[^ ^83^ ^]^), has propelled research toward developing in vitro models that closely mimic human conditions and skin pathologies, as well as being applicable for skin corrosion and irritation testing in accordance with the Organization for Economic Cooperation and Development (OECD) Test Guidelines 431^[^ ^84^ ^]^ and 439,^[^ ^85^ ^]^ respectively.^[^ ^3^, ^86^ ^]^ This highlights the importance of developing precise in vitro models that can faithfully mimic these intricate processes, providing valuable platforms for mechanistic studies and therapeutic development.

## 4 Models to Assess Inflammatory Skin Pathologies

A wide array of engineered skin models has been developed to explore inflammatory conditions and the skin‐immune cells dynamic interaction, supporting controlled and reproducible analysis of inflammatory responses without reliance on animal models. In this context, two types of models have been created: immunogenic skin models that are designed to stimulate an immune response,^[^ ^87^ ^]^ and immunocompetent skin models that incorporate one or more immune cell types, ensuring active and functional involvement of the immune component in the model, being able to stimulate and simulate immune responses.^[^ ^88^ ^]^ **Table** **2** presents a comparative overview of skin models described in the literature that have been used to study inflammatory conditions, emphasizing their cellular composition, materials utilized, type of immune capability (Immunocompetent‐ImC‐ or Immunogenic‐ImG‐), preparation technique, and main outcome.

**Table 2:** Summary of in vitro models found in the literature to study inflammatory conditions in the skin.

Disease or condition	Material	Cells used	Type of immune capability	Preparation Technique	Main Outcome	Ref.
Inflammation	Bovine collagen I Rat tail collagen I	HDFs HEKs Macrophages (buffy coat)	ImC	Manual cast	Application of Bifonazole in the equivalents showed anti‐inflammatory properties in UVB‐ and histamine‐induced skin disorders.	[89]
		HDFs HEKs‐HaCat THP‐1	ImC		The model allowed the performance of transcriptomic analysis and the suitability to study key inflammatory markers after LPS application.	[90]
		HDFs HEKs Monocytes	ImC		Skin differentiation media promote the M2 phenotype and inhibit the M1 phenotype. Macrophage medium hampers skin maturation.	[30]
		SF1‐HDFs Ker‐CT‐cells CD4^+^ T cells PBMCs	ImC		A model infected with *Candida albicans* showed that the application of T6030504 blocks TLR4 activity, exhibiting potential anti‐infective effects.	[91]
	Bovine collagen I + Silk	HDFs HEKs hiNSCs Pre‐adipocytes Endothelial cells Smooth muscle cells Macrophages	ImC	Manual cast	The innervated model enables long‐term culture, supporting immune and neuronal studies with stable pro‐inflammatory factors.	[92]
	–	HDFs HEKs ADSCs BM‐MSCs	ImG	Self‐assembly	The application of probiotic EVs derived from *Lactobacillus paracasei* presented anti‐inflammatory, antioxidant, and antiaging activities in the model.	[93]
		HDFs HEKs Multiple immune/endothelial dermal cells	ImC		A fully autologous model was created. The addition of LPS induced inflammation, keeping the activity of the immune cells.	[94]
	GelMA + HAMA	HDFs HEKs‐HaCat	ImG	Fully bioprinted	BMDA treatment significantly reduced oxidative stress and downregulated pro‐inflammatory cytokines in treated organotypic skin models.	[95]
	SilkMA + GelMA + PPR	HDF HEK HUVEC THP‐1	ImC		The immunocompetent model successfully elicited an inflammatory response, enabling the classification of skin irritants and non‐irritants similar to native skin.	[96]
Psoriasis	Bovine collagen I	HDFs HEKs‐HaCat THP‐1	ImC	Manual cast	Strong macrophage involvement in inflammatory signaling, useful for studying conditions like psoriasis.	[90]
		HEKs HDFs T cells	ImC		Fisetin application exerted pro‐differentiative, antiproliferative, and anti‐inflammatory effects.	[97]
		HDFs HEK Psoriatic peripheral blood	ImC		The incorporation of patient‐specific T cells determined that these cells can infiltrate the epidermis, recapitulating the psoriatic immune response in the skin.	[98]
		HDFs HEK	ImG		The psoriatic model stimulated with Th17 cytokines mimics accurately the psoriatic phenotype and increased AMP expression in the epidermis after Th17 cytokine stimulation.	[99]
	ECM	Psoriatic HDFs Psoriatic HEKs	ImG	Self‐assembly	The patient model mimicked key psoriasis characteristics, and the topical application of tazarotene allows the absorption and metabolization of tazarotene, a psoriatic treatment.	[100]
		Psoriatic HDFs Psoriatic HEKs T cells (Th1 and Th17)	ImC		EPA supplementation normalized the proliferation of psoriatic keratinocytes and decreased the number of IL‐17A‐producing T cells	[101]
		Psoriatic HDFs Psoriatic HEKs T cells	ImC		The addition of ALA to psoriatic equivalents modulated the proliferation of psoriatic keratinocytes and reduced the inflammation and migration of T cells	[102]
		HDFs HEKs	ImG		The model created expressed psoriatic markers when it was stimulated with a Th1 cytokine mix, resulting in an increase in expression of antiapoptotic markers, which prevented psoriatic keratinocytes from death.	[103]
		HDFs HEKs	ImG		The model resembled native skin morphology and met the OECD TG439 standard, making it suitable for irritation testing and disease modeling	[104]
		Psoriatic/Healthy HDFs Psoriatic/Healthy HEKs T cells	ImC		The use of psoriatic cells kept the disease phenotype, and the addition of T cells increased the inflammatory phenotype that was suppressed when methotrexate was applied.	[105]
		Psoriatic/Healthy HDFs Psoriatic/Healthy HEKs	ImG		Application of DHA reduced the inflammation and restored normal keratinocyte differentiation and proliferation	[106]
		Psoriatic/Healthy HDFs Psoriatic/Healthy HEKs T cells PBMCs	ImC		The addition of activated T cells to the model enhanced IL‐17A production and replicated key features of native psoriatic skin that were downregulated after anti‐IL‐17A treatment.	[40]
		HDFs HEKs Th1 cells	ImC		Th1 cells changed the expression of epidermal barrier proteins and exhibited functional immune activation.	[99]
		HDFs HUVECs HEKs‐HaCat T cells (Th17)	ImC	Partially bioprinted	Incorporation of HUVECs enables the creation of a vascularized psoriatic model that, upon IL‐22 and TNF‐α stimulation, expresses characteristic psoriatic factors, which are suppressed following treatment with tofacitinib and Danshensu	[107]
Atopic Dermatitis	ECM	HDFs HEKs	ImG	Self‐assembly	Th2‐driven cytokines reduced the expression of differentiation markers and caused defects in the epidermal barrier.	[103]
		HDFs HEKs	ImG		Inhibited AMP expression in skin equivalents stimulated with Th2 cytokines increases the susceptibility to *S. aureus* colonization.	[99]
	Rat tail Collagen I	HDFs HEKs	ImG	Manual cast	Osthole, a plant‐derived compound, improved the skin barrier and reduced inflammation.	[108]
		HDFs HEKs	ImG		Osthole, a plant‐derived compound, inhibited the Th2 cell response and reduced the production of IL‐4 and IL‐13.	[109]
	Fibrinogen + Novogel component 2	HDFs HEKs iPSC endothelial Human placental microvascular pericytes	ImG	Partially bioprinted	The model replicated skin morphology and barrier function. The endothelial cells enhanced chronic inflammation, allowing the model to be used for accurate drug screening.	[110]
Diabetes	ECM from decellularized porcine dermis, hypodermis, and vascular tissue	Human healthy/diabetic HDFs Human healthy/diabetic subcutaneous preadipocyte cells HEKs HUVECs	ImG	Fully bioprinted	The model replicated key pathophysiological features of type 2 diabetes in the skin. The drug testing showed accuracy in the reduction of inflammatory factors.	[28]
	Porcine Collagen I	HDF dHDF HEK Diabetic Macrophages	ImC	Manual cast	The interaction of dHDF with the macrophages induced the polarization to the M1 phenotype and an increase in the secretion of proinflammatory factors	[75]

ImC: Immunocompetent; ImG: Immunogenic; HDF: Human Dermal Fibroblast; HEK: Human Epidermal Keratinocytes; Th: T helper cell; ADSCs: Adipose Stem Cells; BM‐MSCs: Bone Marrow Stem Cells; PBMCs: Peripheral blood mononuclear cells; LPS: Lipopolysaccharide; ECM: Extracellular Matrix. HUVECs: Human umbilical vein endothelial cells; LPS: Lipopolysaccharide; IMQ: Imiquimod; TLR4: Toll‐like Receptor 4; EVs: Extracellular vesicles; HAMA: Hyaluronic acid methacrylated; BMDA: N‐Benzyl‐N‐methyl‐dodecan‐1‐amine; PPR: Photoactivated platelet releasate; AMP: Antimicrobial peptides; EPA: Eicosapentaenoic acid; ALA: alpha‐linolenic acid; DHA: docosahexaenoic acid; dHDF: diabetic Human Dermal Fibroblasts.

### 4.1 Use of Commercial Skin Models to Assess Skin Inflammatory Conditions

The first skin model developed after the conventional 2D primary cultures and cell lines was the reconstructed human epidermis (RHE). In the 1990s, commercial RHE models such as EpiSkin (developed by L'Oréal) and EpiDerm™ (by MatTek) emerged and were subsequently validated for use in skin irritation and corrosion testing, offering reliable alternatives to animal testing in line with regulatory guidelines.^[^ ^78^, ^111^ ^]^ This model involves seeding keratinocytes on a polycarbonate membrane and then culturing them under air‐liquid interface (ALI) conditions to create a fully differentiated epidermis.^[^ ^112^ ^]^ However, in recent years, the role of fibroblasts in the dermal compartment concerning morphogenesis, phenotype of keratinocytes, and maintenance of skin structure in both health and disease has gained increasing attention.^[^ ^63^, ^113^ ^]^ This growing understanding has driven the development of full‐thickness (FT) in vitro skin models to improve physiological relevance by replicating the structural and functional complexity of native human skin.^[^ ^78^, ^114^ ^]^ As summarized in Table 2, a range of inflammatory skin pathologies have been successfully modeled using FT‐skin systems over the past six years, and several commercially available FT skin models have been developed and used to investigate inflammatory skin conditions, as is summarized in **Table** **3**. These commercial platforms provide the flexibility to integrate immune components, enabling the creation of immunocompetent or immunogenic skin models. Such systems are valuable for assessing substance toxicity,^[^ ^115^ ^]^ facilitating drug discovery,^[^ ^116^, ^117^ ^]^ and evaluating the effects of various secretomes on skin physiology and immune responses (Table 2).^[^ ^118^ ^]^

**Table 3:** Commercial models and their application in the study of inflammatory skin diseases.

Commercial model	Composition	Disease or condition	Main outcome	Ref.
**Labskin^TM^** Labskin‐S	HDF HEK	–	The model can be colonized by skin commensals like *S. epidermidis*, and allow the testing of natural components used in skin care.	[115]
**MatTek** SOR‐300‐FT‐CTR	Psoriatic HDF HEK	Psoriasis	The model used validated the role of IL‐36γ in psoriasis and was also useful in testing the biological activity of an IL‐36γ antagonist, A‐552, attenuating the disease markers.	[117]
			The study highlighted the model's utility as a translational preclinical platform for studying psoriasis and testing targeted therapies.	[116]
**Phenion** Full‐Thickness (FT) Skin Model;	HDF HEK	Acne	The model was complemented with the T cells. It was shown that Myrtacine reduced *C. acnes* virulence factors, while Celastrol‐enriched extract (CEE) inhibited IL‐17A production.	[53]
**Lab Skin Creations** Full‐thickness human 3D skin model	HDF HEK	Inflammation	The model demonstrated that an endothelial cell‐derived inflammatory secretome can induce a sustained pro‐inflammatory fibroblast phenotype without immune cell involvement.	[118]

Commercially available organotypic skin models, along with those developed in‐house, have extensive applications in clinical, research, and industrial settings. These models represent a promising alternative in response to regulatory restrictions on animal testing. Emerging technologies, particularly 3D bioprinting, have the potential to significantly enhance the accessibility and functional complexity of engineered skin models. Collaborative efforts, such as those between L'Oréal and the University of Oregon, are exploring advanced fabrication methods, including melt electrowriting, to produce biomimetic artificial skin with structural and functional properties closely resembling native skin.^[^ ^14^, ^119^ ^]^ In regenerative medicine, companies like Poietis have developed 3D‐bioprinted skin grafts and printers aimed at improving wound healing outcomes.^[^ ^120^ ^]^ Similar initiatives are gaining attention, particularly within the cosmetics industry, where businesses across Asia, Europe, and South America are actively developing proprietary 3D‐bioprinted skin models.^[^ ^14^ ^]^ These efforts are designed not only for product testing but also for potential therapeutic applications in regenerative medicine.^[^ ^14^ ^]^

### 4.2 Approaches for the Development of Skin Equivalents in the Study of Inflammatory Conditions

**Figure 3:**
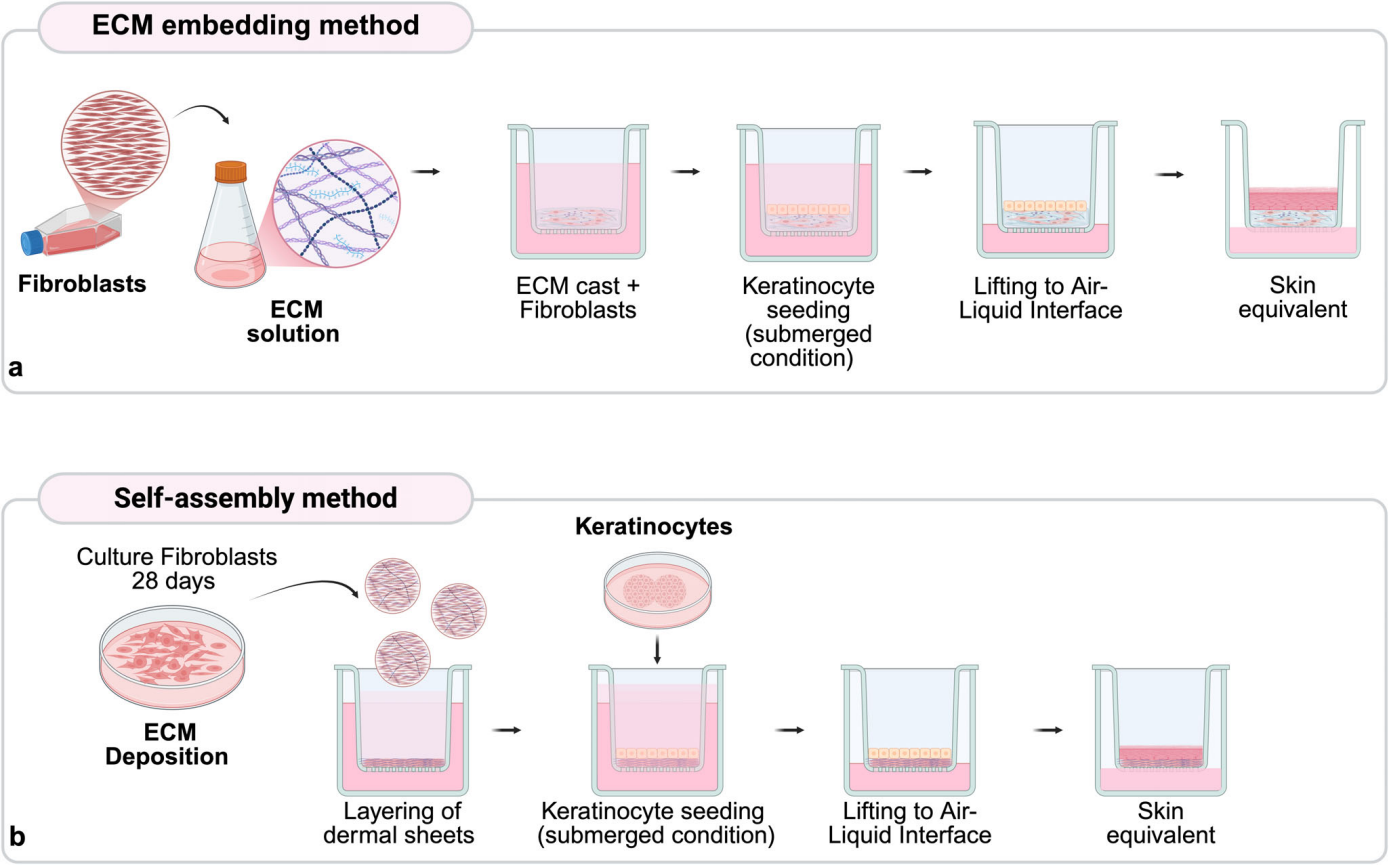
Manual strategies to develop in vitro skin models **a) ECM embedding method**: the dermal compartment is formed by the embedding of fibroblasts in collagen or any other ECM component and cast in transwell inserts, keratinocytes are seeded on top, followed by culture in ALI conditions to promote epidermal differentiation. **b) Self‐assembly method**: the dermal compartment is formed by the deposition of ECM by fibroblasts cultured for 28 days in ascorbic acid‐supplemented medium; the sheets created are layered in transwell inserts, and keratinocytes are seeded on top, followed by culture in ALI conditions to promote epidermal differentiation. In both cases, final organotypic skin models show stratified epithelia. Created in BioRender. Ulloa, A. (2025) https://BioRender.com/lmhudev.

The most employed approaches are shown in **Figure** **3** and include the embedding of fibroblasts within various extracellular matrix components, the self‐assembly method (also referenced in Table 2), and several advanced 3D bioprinting techniques discussed in the next sections.

The ECM embedding methods involve mixing fibroblasts in a solution of natural biomaterials, such as collagen I derived from bovine or rat tail, fibrin, chitosan, or gelatin^[^ ^78^, ^121^ ^]^ (Figure 3a). These mixtures are cast in transwell inserts containing a synthetic membrane that supports the entire structure, followed by keratinocyte seeding and ALI culture conditions for keratinocyte differentiation.^[^ ^122^ ^]^ As a major component of the native skin ECM, collagen I is widely used in the biofabrication of organotypic skin models due to its biocompatibility and ability to enhance complexity, including vascularization. However, collagen‐based organotypic skin models exhibit low mechanical strength and tend to contract, which increases the degradation rate in vitro,^[^ ^92^ ^]^ shortening the lifespan of the equivalents.

The self‐assembly method was developed to minimize the immunogenicity of synthetic membranes in regenerative medicine but was later applied to create in vitro skin models for research^[^ ^123^, ^124^ ^]^ (Figure 3b). This approach involves seeding fibroblasts in cell culture media enriched with ascorbic acid to enhance the secretion of endogenous extracellular matrix for 28 days.^[^ ^122^ ^]^ The resulting sheets can be layered, and keratinocytes can be seeded on top of this construct, and then these equivalents are raised to ALI to promote epidermal differentiation (Figure 3b).

Among the broad spectrum of inflammatory skin conditions modeled in vitro, inflammation, psoriasis, atopic dermatitis, and diabetes are among the most extensively studied (Table 2). For broad inflammatory responses, several immunocompetent skin models have been developed using mainly manual techniques. These incorporate macrophages as a standard strategy to mimic a broad inflammatory response. As part of the innate immune response, macrophages can present pro‐ or anti‐inflammatory phenotypes, according to signals and triggers in the microenvironment.^[^ ^125^ ^]^ Inflammatory skin models enable the assessment of the anti‐inflammatory effects of compounds such as bifonazole, an antifungal agent.^[^ ^89^ ^]^ Furthermore, they enhance the transcriptomic sensitivity of data collection through the interaction between keratinocytes and macrophages. The models mimic the innate immune response in the presence of different challenges and enabling understanding of the influence of proinflammatory factors in wound healing, UV damage response, and the detailed examination of the mechanisms involved.^[^ ^90^ ^]^ In summary, the macrophage‐based construct is a simple yet reproducible model that captures the fundamental innate immune response. It facilitates mechanistic studies and compound testing with physiological fidelity and long‐term stability, although further research is required to investigate this in more detail. Compared to these specific models, more complex approaches, like hypodermis and nervous system inclusion, together with immune components, like the model presented by Vidal et al, provide important insights about neuroimmune interactions in skin inflammatory pathologies.^[^ ^92^ ^]^

Furthermore, the works presented by Vidal et al.,^[^ ^92^ ^]^ Griffoni et al.,^[^ ^30^ ^]^ and Attiogbe et al. ^[^ ^94^ ^]^ demonstrate significant elements that must be considered when creating immunocompetent skin models. Vidal et al. demonstrate that the utilization of collagen as a matrix for the creation of in vitro skin models is accompanied by the disadvantage of contraction, thereby limiting the application and duration of the models. To address this issue, the use of alternative biomaterials, such as silk, is proposed. This approach resulted in the creation of skin models whose dimensions remained consistent for ≈40 days, thereby facilitating the exploration of the immune response in chronic skin diseases.^[^ ^92^ ^]^ Additionally, Griffoni et al. highlighted the importance of keeping a balanced cell culture medium to preserve phenotype and viability in multicellular skin models. In an immunocompetent model with macrophages, it has been demonstrated that the use of macrophage medium has the effect of impairing epithelial differentiation and reducing keratinocyte viability; and the use of epithelial differentiation medium has been shown to reduce the secretion of pro‐inflammatory factors in macrophages M1, promoting a M2 phenotype.^[^ ^30^ ^]^ It was also discussed by Attiogbe et al. that the use of a poor medium with minimal factors is to be recommended, based on the hypothesis that the same microenvironment will produce the necessary components to keep and support the multicellular scaffold. In a study employing a fully autologous model, it was demonstrated that immune cells retained the capacity to exhibit inflammatory activity after being challenged with LPS.^[^ ^94^ ^]^ These works demonstrate how the selection of scaffold and medium directly influences the duration of the study and the immune activity, with strategies ranging from balanced to minimalistic supplementation. Such approaches enhance physiological relevance; however, they concomitantly introduce variability and technical challenges.

In addition to manual techniques, 3D bioprinting has been used to model skin inflammation as a platform to assess, for example, the anti‐inflammatory potential of N‐Benzyl‐N‐methyl‐dodecan‐1‐amine (BMDA) derived from garlic extracts. The research conducted by Khang et al. demonstrates the development of an immunogenic bioprinted model that secretes various proinflammatory factors in response to H_2_O_2_ challenge, followed by subsequent suppression upon the addition of BMDA.^[^ ^95^ ^]^ It can be suggested that the biofabrication of a bilayer model does not constitute an increase in complexity; nevertheless, the work indicates the use of alternative natural biomaterials in its creation, which have enhanced its mechanical properties and printability, similar to native tissue with special control in the cell deposition, and as a clear advance over the biomaterials used in manual techniques. As will be discussed in later sections, bioprinting techniques have been used to increase the complexity of tissue models, as demonstrated in the work presented by Bhar et al.^[^ ^96^ ^]^ The authors present a high‐throughput platform for preclinical skin sensitization assessment in compliance with regulatory guidelines. Furthermore, the preparation of the model involves the addition of macrophages and endothelial cells, thus enhancing both its complexity and its physiological relevance.^[^ ^96^ ^]^ Unlike manual models, 3D bioprinting provides spatial control, architecture reproducibility, and the use of advanced biomaterials. However, there are ongoing questions about standardization across different systems. T cells have also been incorporated into in vitro skin models and combined with microorganisms or derivatives of the skin microbiome to support the investigation of immune‐modulating therapies in inflammation, including the characterization of novel TLR4 antagonists by Merk et al., where they use a *Candida albicans*‐infected skin model that also contains CD4^+^ T cells. In this model, they show that the infection of an immunocompetent model with *C.albicans* can trigger inflammation, which can be suppressed when TLR4 agonists are applied to the model, showing their potential anti‐infective.^[^ ^91^ ^]^ Furthermore, immunogenic models have been used to evaluate the effects of probiotics contained in extracellular vesicles on skin physiology^[^ ^93^ ^]^ (Table 2). The application of pathogen and microbiome‐based models has the potential to extend immunocompetence beyond the scope of innate immune responses, thereby demonstrating interactions between T‐cells and microbes. Although these findings are highly relevant to infection‐driven inflammation, it should be noted that these are specialized models with limited generalization and higher variability.

Diverse immune cell populations and cytokine cocktails have been used to create both immunocompetent and immunogenic psoriatic skin models (Table 2). These include the use of THP‐1, a commercially available macrophage cell line, as well as peripheral blood mononuclear cells (PBMCs) and T cells activated or polarized to Th1 and Th17 phenotypes. Cho et al. reported that the application of imiquimod, a synthetic TLR7 agonist that can be used to induce psoriasis in animal models, triggers a strong pro‐inflammatory response in macrophages.^[^ ^90^ ^]^ Nevertheless, the absence of T cells in the model hinders the ability to determine whether the macrophages and imiquimod stimulus are sufficient to replicate the psoriatic pathology, even though there is a clear increase in IL‐23 gene expression, but not histological characterization of psoriatic markers or secretion of proinflammatory cytokines typically found in psoriasis.

The therapeutic potential of various bioactive molecules and dietary compounds has been assessed using these models. Chamcheu et al. established a model using the manual approach with collagen and adding T cells isolated from PBMCs and activated with CD3/CD28. The activated T cells in the model enhance the expression of psoriasin and the secretion of IL‐17A, key hallmarks of a psoriatic phenotype. When the equivalents were treated with fisetin, a phytochemical found in colored fruits and vegetables, it improved the psoriatic phenotype by inhibiting key metabolic pathways.^[^ ^97^ ^]^ Morin et al. established a self‐assembled and autologous model taking fibroblasts and keratinocytes from healthy and psoriatic patients, then applied tazarotenic acid^[^ ^100^ ^]^ or docosahexaenoic acid (DHA),^[^ ^106^ ^]^ demonstrating that cells from psoriatic patients retained the disease phenotype under in vitro culture conditions, while the adding of the different compounds reduced the expression of psoriatic features, and identifying the signaling mechanisms involved.^[^ ^100^ ^]^ Specifically, DHA was shown to rebalance the expression of PPAR receptors, leading to decreased TNF‐α secretion and a reduction in psoriatic characteristics.^[^ ^106^ ^]^ The same research group developed various models incorporating T cells polarized to Th1 or Th17 lineages^[^ ^105^ ^]^ and found that eicosapentaenoic acid (EPA) suppressed IL‐17A release,^[^ ^101^ ^]^ while alpha‐linolenic acid (ALA) reduced T cell activation signaling pathways and overall inflammation.^[^ ^102^ ^]^ These models also offered insights into the crosstalk between psoriatic keratinocytes and T cells.^[^ ^40^ ^]^ In support of this, Shin et al. and Lorthois et al. ^[^ ^98^, ^105^ ^]^ demonstrated that this interaction is facilitated by T cell infiltration from the dermal to the epidermal compartment (Table 2).

Additionally, Scheurer et al. and Morgner et al. demonstrated that both immunogenic and immunocompetent models replicate the histological and functional characteristics of native psoriatic tissue, a pattern also seen in models of atopic dermatitis.^[^ ^99^, ^103^ ^]^ Importantly, even the isolated stimulation of skin models with IL‐17A alone can trigger changes that resemble psoriatic pathology.^[^ ^104^ ^]^ In parallel, the use of 3D bioprinting techniques has been explored for psoriasis modeling. Peng et al. developed an immunocompetent and vascularized psoriatic model as a platform for drug testing. In this model, apart from Th17 cells, there is an additional activation with IL‐22 and TNF‐α, to enhance the psoriatic characteristics, which lead to epidermal hyperplasia and the secretion of inflammatory cytokines, a phenotype that is suppressed after pharmacological treatment with tofacitinib and danshensu.^[^ ^107^ ^]^

These examples illustrate the variability in the protocols for incorporating immune cells into psoriatic skin models. It is well established that psoriasis is primarily a Th17‐driven skin disease. However, many models employ macrophages or CD3/CD28‐activated T cells, which promote extensive proinflammatory responses (e.g., IFN‐γ, TNF‐α, IL‐2) yet fail to encompass the IL‐23/IL‐17 axis and coordinate activation of Th17 cells, a pivotal component of psoriasis.^[^ ^126^ ^]^ In contrast, autologous or Th17‐polarized models exhibit a higher degree of fidelity in reproducing disease‐specific mechanisms, including IL‐17A secretion and keratinocyte T cell crosstalk. However, these models also introduce increased complexity, variability, and scalability challenges. This comparison highlights a key trade‐off: reductionist models offer control and reproducibility for compound testing, while more elaborate systems capture pathophysiological specificity at the cost of practicality.

AD has been extensively modeled in vitro, primarily through the development of immunogenic skin organotypic skin models using manual techniques and, in recent years, 3D bioprinting. These models are typically used to simulate the disease by exposing reconstructed skin to Th2 cytokine cocktails or molecules such as histamine and LPS that mimic the pathophysiological features of AD.^[^ ^103^ ^]^ It has been demonstrated that they possess significant value in the evaluation of drug candidates. In this context, Kordulewska et al. have demonstrated that osthole, a naturally occurring coumarin, exerts immunomodulatory activity in in vitro models of AD prepared using the collagen manual casting technique, stimulated with histamine and LPS. The immunomodulatory activity of osthole has been shown to be associated with the toll‐like receptor 2 (TLR2) signaling pathway. The downregulation of TNF receptor‐associated factor 6 (TRAF6) and IL‐4 α receptor has been demonstrated to improve the integrity of the functional barrier and reduce inflammation.^[^ ^108^, ^109^ ^]^ The approach of Scheurer et al. ^[^ ^99^ ^]^ and Morgner et al. ^[^ ^103^ ^]^ to produce AD in vitro models is based on a self‐assembly technique. The consensus of both works is that the scaffolds generated by self‐assembly have a longer lifespan (up to three months) than the collagen skin equivalents (maximum two weeks).

Scheurer's research demonstrates a heightened susceptibility to *Staphylococcus aureus* colonization in AD models, attributable to a decline in AMP expression.^[^ ^99^ ^]^ Morgner et al. furthermore argue the use of this approach on the basis that the ECM secreted by the fibroblasts produces a dermal compartment enriched with ECM components that can better mimic pathological scenarios than other approaches and studies. Additionally, they highlight that in a model stimulated with cytokines that are typically added to the medium below, the transport of the cytokines to the epithelium can be challenged in collagen skin equivalents, impeding the proximal regulation.^[^ ^103^ ^]^

Moreover, the integration of 3D bioprinting technologies has facilitated the incorporation of vasculature, as demonstrated by Liu et al. They created a vascularized skin model for AD as a high‐throughput platform for drug screening, while also comparing it with a non‐vascularized model.^[^ ^110^ ^]^ In the vascularized model, the hallmarks of AD are more pronounced. The application of IL‐4 has been shown to reduce angiogenesis and affect the integrity of the barrier function to a higher degree than in the non‐vascularized models. However, the application of dexamethasone and JAK inhibitors has enhanced the recovery of the barrier function and improved epidermal differentiation. This demonstrates that increasing structural and cellular complexity can enhance the fidelity and responsiveness of AD disease models.^[^ ^110^ ^]^

One of the main challenges in the development of 3D skin models is the integration of 3D printing technologies alongside dynamic immune components, particularly in the context of drug development for inflammatory skin diseases. Notably, advances in 3D bioprinting technology have facilitated the construction of complex, multicellular skin models that assist the study of systemic conditions such as diabetes.^[^ ^28^ ^]^ For example, Smith et al., in a manually cast model, show the role of the diabetic fibroblast on the macrophage polarization toward a proinflammatory M1 phenotype.^[^ ^75^ ^]^ In contrast, Kim et al applied extrusion‐based 3D bioprinting to generate a vascularized and perfusable FT skin model with hypodermis replicating key features of type 2 diabetes. They showed how vascular dysfunction, adipose hyperplasia, and proinflammatory response take place. Importantly, their platform enabled drug testing that demonstrated the restoration of epidermis and downregulation of the inflammatory response.^[^ ^28^ ^]^

Combining both approaches into a complex model like the one presented by Kim et al. could provide valuable insights into the immune crosstalk of M1 macrophages in processes such as angiogenesis, wound healing, and ECM remodeling, as well as their response to immunomodulatory drug treatments.^[^ ^28^ ^]^ Moreover, such an integrated model could offer a novel opportunity to investigate the immunopathology of conditions like vitiligo, hidradenitis suppurativa, and chronic spontaneous urticaria, for which there is still a lack of in vitro models that fully recapitulate both skin architecture and immune dysfunction.

## 5 Application of 3D Bioprinting in the Development of Skin Models

3D bioprinting is a cutting‐edge additive manufacturing technology used to produce volumetric objects with complex geometries based on a computer‐aided design (CAD) model.^[^ ^78^ ^]^ This involves depositing layers of living cells embedded in a material of interest, one layer at a time.^[^ ^127^, ^128^ ^]^ The cell‐laden biomaterial is called bioink.^[^ ^129^ ^]^ For skin tissue engineering applications, creating an in vitro model that replicates complex interactions and architectures present in native skin and discussed in previous sections still have major challenges, where 3D bioprinting can offer solutions.^[^ ^130^, ^131^ ^]^

3D bioprinting offers several advantages in the development of skin models over the manual techniques, which are summarized in **Table** **4**. Advantages include precision in controlling the shape and the spatial distribution of the ink within the extruded construct.^[^ ^121^ ^]^ This enables precise control of cell distribution.^[^ ^78^, ^131^, ^132^ ^]^ This level of control enables the possibility of increasing the complexity of the organotypic skin models to meet specific characteristics. In the development of skin disease models, the absence of appendages and immune system has limited the improvement of in vitro models and drugs for conditions that affect these structures.^[^ ^129^ ^]^ The use of multiple print heads available in 3D printers allows the increase of cell heterogeneity and the addition of skin appendages, like sweat glands, nerves, hair follicles, or strategically adds specific immune cell subsets. Additionally, it improves the replication of native tissue architecture, contributing to the creation of physiologically relevant skin models.^[^ ^78^, ^132^, ^133^ ^]^ Because 3D bioprinting is more automated than manual techniques for fabricating organotypic skin models, it allows scalability and improved quality control throughout the process. It also provides greater reproducibility for high‐throughput applications such as drug testing or the production of large‐size equivalents for regenerative medicine.^[^ ^78^, ^120^, ^134^ ^]^ The pathologies described in section 3 show specific requirements due to the anatomical structures and immunological responses involved. 3D bioprinting techniques can address these requirements, offering alternative models but also transformative tools that allow the design of skin equivalents according to the studied disease.

**Table 4:** Advantages and disadvantages of manual and bioprinting techniques in the manufacturing of skin models.

Manual techniques	Bioprinting
Advantages: – Cost‐effective and easy implementation – Low immune activation due to the use of ECM derivatives Disadvantages: – Limited scalability and number of replicates – Poor reproducibility batch to batch – Limited increase in complexity – Limited lifespan	Advantages: – Precision – Easy implementation – ↑ complexity and tissue architecture – Scalability and possibility to produce large‐size equivalents – Better reproducibility – Advances in biomaterials development increase the lifespan of the equivalents Disadvantages: – High operational costs (advanced instrumentation and expert handling) – Immunological interaction of the materials used

Although 3D bioprinting shows promise, technical and biological concerns must be addressed to develop immunocompetent skin models. One disadvantage arises when biomaterials are mixed with synthetic materials to make them more printable. When used in 3D bioprinting for wound healing applications, they can induce immune cell activation. In the development of in vitro immunocompetent models, they could activate immune cells in the model.^[^ ^134^ ^]^ The biomaterials used in manual techniques are extracellular matrix (ECM) derivatives that usually lack cellular antigens that can activate the immune cells in a model.^[^ ^135^ ^]^

**Figure 4:**
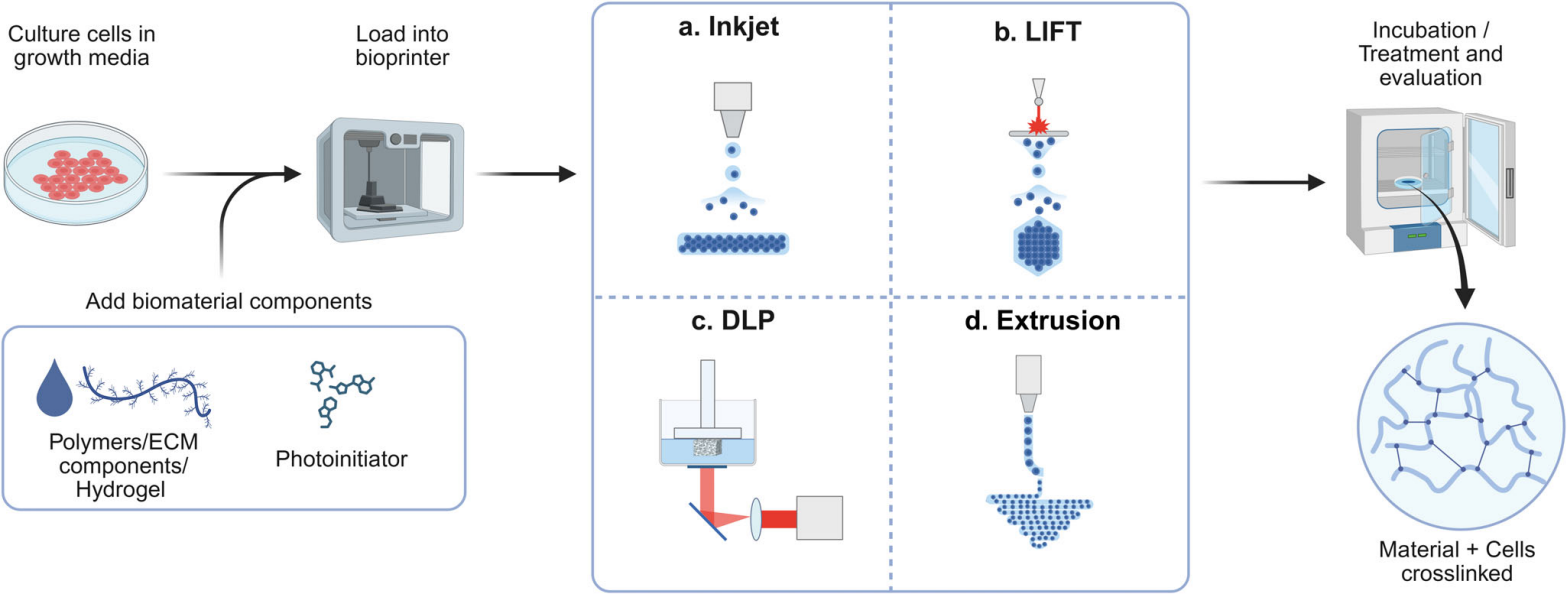
Schematic representation of bioprinting techniques used in the manufacturing of organotypic skin models. The basic workflow consists of mixing the cells with the biomaterial, which may include a photoinitiator in light‐based bioprinting techniques, according to the bioink formulation to be printed by **a) Inkjet‐based bioprinting, b) Laser‐induced Forward Transfer (LIFT), c) Digital Light Processing** (DLP), or **d** **) Extrusion‐based bioprinting**. Once the organoypic models are printed and crosslinked according to the bioink, they are incubated for further analysis. Created in BioRender. Ulloa, A. (2025) https://BioRender.com/535eekh.

The most common ECM derivative used in skin models is the bovine or rat tail collagen I, which is mixed with fibroblasts.^[^ ^121^ ^]^ The lack of additional interactions and cross‐linking with other ECM components results in stronger contractile forces in the fibroblasts and poor mechanical strength, causing them to shrink the dermal compartment and decrease the volume and area of the entire skin equivalent.^[^ ^121^ ^]^ This limits the equivalent's lifespan. The development of 3D bioprinting technologies has allowed the creation of a wide range of biomaterials, primarily for extrusion‐based techniques. Many of these address this issue by increasing the mechanical properties and thus the lifespan of the organotypic skin models. Several strategies, along with a variety of biomaterials that possess different properties, will be discussed in the following sections. The most used techniques employed for bioprinting organotypic skin models include inkjet bioprinting, extrusion‐based bioprinting, and light‐based bioprinting; these can be used independently or in combination with other methods (**Figure** **4**). **Table** **5** provides an overview of the progress in bioprinted skin models, showing representative 3D bioprinted models developed in the last 6 years. The period was chosen to highlight recent innovations, particularly those aiming to improve structural complexity. While technological advances have significantly enhanced the mimicking of native tissue, one limitation remains: the lack of an immune system. To highlight this gap, we include the “immune capability” column, which refers to whether the models include immune cells or cytokine stimulation. Most of the presented models lack this feature, emphasizing the need to integrate immunocompetence in future developments.

**Table 5:** Overview of bioprinted skin models (2019‐2025). Advances in structural complexity for different applications.

Skin model type	Layers represented	Cell types incorporated	Bioink(s) used	Bioprinting technology	Complexity	Immune capability	Application	Ref.
Reconstructed human epidermis	Epidermis	HEK	–	Inkjet	Low	No	DM: atopic dermatitis and ichthyosis vulgaris	[136]
Reconstructed human dermis	Dermis	HDF Melanocytes	Decellularized ECM from porcine dermis and epidermis	Extrusion	Low	No	DM: Metastatic melanoma. Drug testing	[137]
Reconstructed human dermis	Dermis	HDF Myofibroblasts	–	LIFT	Low	No	Tissue modeling and wound healing	[138]
Reconstructed human dermis vascularized	Dermis	HDF HUVECs	GelMA/HA‐NB/LAP	DLP	Low	No	Tissue modeling and wound healing	[139]
Full thickness	Dermis Epidermis	HDF PBMCs HEK	– –	Inkjet	Low	Yes	Drug testing	[140]
	Dermis Epidermis	HDF HEK	– –	LIFT	Low	No	Regenerative medicine	[120]
	Dermis Epidermis	HDF HEK	Porcine collagen I –	Inkjet Microextrusion	Low	No	Tissue modeling	[141]
	Dermis	HDF	Gelatin‐Fibrinogen‐Collagen I‐ Elastin	Extrusion	Low	No	Drug testing	[142]
	Basement Membrane	–	Laminin‐Entactin					
	Epidermis	HEK	–					
	Dermis Basement Membrane Epidermis	HDF – HEK‐HaCat	Collagen I / Fibrinogen	Extrusion	Low	No	Tissue modeling	[143]
	Dermis Epidermis	HDF HEK	Alginate‐ gelatin‐diethylaminoethyl cellulose	Extrusion	Low	No	Tissue modeling	[144]
	Dermis Epidermis	HDF HEK	Alginate‐ gelatin‐diethylaminoethyl cellulose	Extrusion	Low	No	Tissue modeling	[145]
	Dermis Epidermis	HDF HEK	Gelatin‐alginate‐fibrinogen	Extrusion	Low	No	Tissue modeling	[146]
	Dermis Epidermis	HDF HEK	Collagen	Extrusion	Low	No	Tissue modeling	[147]
	Dermis Epidermis	HDF HEK	GelMA‐ Bacterial nanocellulose	Extrusion	Low	No	Tissue modeling	[148]
	Dermis Epidermis	HDF HEK‐ HaCat	GelMA+ HAMA	Extrusion	Low	No	Drug testing	[95]
	Hypodermis Dermis Epidermis	MSC HDF HEK	Collagen I	Extrusion	Medium	No	Tissue modeling	[149]
	Dermis Epidermis	HDF HEK	Collagen I	Inkjet	Low	No	Regenerative medicine	[150]
	Hypodermis Dermis Epidermis	SVF HDF HEK	Collagen‐pectin	Extrusion	Medium	No	Tissue modeling	[151]
Vascularized‐ full thickness	Dermis Epidermis	HDF Human iPSC endothelial cells Human placental microvascular pericytes HEK	Fibrinogen + Novogel component 2	Extrusion	Medium	Yes	Disease modeling: Atopic dermatitis	[110]
	Dermis Epidermis	HDF HUVECs HEK	Silk‐GMA Gel‐GMA	DLP	Medium	No	Wound healing	[152]
	Dermis Epidermis	HDF HUVECs THP‐1 activated to M0 HEK	SilkMA + GelMA + PPR	Extrusion	High	Yes	Sensitization assessment	[96]
	Dermis Epidermis	HDF HUVECs T cell (Th17) HEK‐HaCat	GelMA+Collagen	Extrusion	Medium	Yes	DM: Psoriasis Drug testing	[107]
Vascularized‐perfusable full‐thickness	Hypodermis Dermis Epidermis	Human healthy/diabetic subcutaneous preadipocyte cells Healthy/diabetic HDF HUVECs HEK	ECM from decellularized porcine dermis, hypodermis, and vascular tissue	Extrusion	High	No	DM: Diabetes type 2 Drug testing	[28]
	Dermis Epidermis	HEK HUVECs Pericytes HEK	Collagen I	Extrusion	Medium	No	Regenerative medicine	[153]
Full thickness with appendages	Dermis Sweat glands Hair follicles Epidermis	HDF Plantar dermis homogenate MSC HEK	Gelatin‐alginate	Extrusion	High	No	Tissue modeling	[154]
Full thickness with appendages, vascularized, and pigmented	Dermis Hair follicles Epidermis	HDF Dermal papilla cells HUVECs HEK Melanocytes	Collagen I Dermatan sulfate Collagen IV	Extrusion	High	No	Tissue modeling	[155]
	Hypodermis Dermis Hair follicles Epidermis	Adipocytes HDF Follicle dermal papilla cells HEK Melanocytes	Fibrinogen‐ gelatin‐glycerol‐hyaluronic acid	Extrusion	High	No	Tissue modeling Regenerative medicine Wound healing	[156]

DM: disease modeling

### 5.1 Inkjet Bioprinting

In this technique, the bioink, which possesses a very low viscosity, is ejected from the printer head drop by drop onto a surface.^[^ ^141^ ^]^ The mechanism for forming bioink tiny droplets is driven by either a thermal or a piezoelectric tool (Figure 4a). This technique offers the advantage of high‐speed printing while maintaining cell viability and featuring low manufacturing costs. However, it may cause cellular damage due to mechanical stress.^[^ ^157^ ^]^ Inkjet bioprinting enables precise layering and special organization in organotypic skin models and has been used to create RHE^[^ ^136^ ^]^ and FT models.^[^ ^140^, ^141^ ^]^

Madiedo‐Podvrsan et al. employed inkjet bioprinting to create an RHE model to study AD and ichthyosis vulgaris (IV) using two halves or concentric circles with two different keratinocyte populations: native keratinocytes and filaggrin knockdown (shFLG) transduced NHKs. The concentric rings approach yielded more physiologically relevant outcomes, as it mimics a dermatological spot, allowing the study of the interface between healthy and diseased tissue, since every cellular type keeps its position over the culture time, allowing the compartmentalization of different cell types in the same structure.^[^ ^136^ ^]^ Lee et al. combined microextrusion with inkjet bioprinting to create an FT model, focusing on the advantages of depositing keratinocytes to form the epidermis using inkjet bioprinting and comparing them with manual techniques.^[^ ^141^ ^]^ They showed that the bioprinting of the keratinocytes improved the uniformity in the epidermal layers, the tissue morphological quality, and reduced the keratinocyte aggregates in comparison with manual techniques. Ahmed et al. applied the same technique to develop a full‐thickness immunocompetent skin model as a platform for therapeutic antibody testing in a 96‐well plate format. The model showed the expression of relevant skin differentiation markers and functional immune response, where the muromonab induced a T‐cell response. This model also serves as a platform to determine the cytokine profile elicited by the immunotoxicity of monoclonal antibodies^[^ ^140^ ^]^ (Table 5).

Together, these studies highlight distinct applications of inkjet bioprinting: Madiedo‐Podvrsan et al. prioritized micro‐scale pathological relevance by spatially patterning healthy and diseased keratinocytes, showing a possible application to studying eczema‐like diseases where there is an impairment in the barrier function; Lee et al. focused on improving tissue architecture and reproducibility by enhancing epidermal uniformity; and Ahmed et al. demonstrated the scalability of full‐thickness, immunocompetent models for high‐throughput therapeutic testing.^[^ ^136^, ^140^, ^141^ ^]^ The trade‐off lies between physiological specificity, structural control, and translational scalability. Overall, inkjet bioprinting increases precision in keratinocyte deposition and layering, improving the quality and consistency of organotypic skin models. Given its adaptability, this methodology could also support disease‐specific applications, such as vitiligo, where the controlled positioning of melanocytes in the basal epidermis would enable the study of immune‐mediated melanocyte destruction in a physiologically relevant microenvironment.

### 5.2 Laser‐Induced Forward Transfer (LIFT)

In LIFT, a laser serves as the actuation source to transfer cell‐laden material onto the deposition platform in the form of a bubble, eliminating the necessity for a nozzle while bioprinting and therefore avoiding shear stress and nozzle clogging^[^ ^158^, ^159^, ^160^ ^]^ (Figure 4b). This method preserves the high viability of the printed constructs while achieving excellent printing resolution. Douillet et al. used LIFT to pattern different ratios of fibroblasts and myofibroblasts on a collagen bed to study the cell dynamics during tissue remodeling and wound healing, with the aim of understanding the dynamic and equilibrium between cell populations to avoid the appearance of hypertrophic scars due to fibrosis.^[^ ^138^ ^]^ Taking a step toward clinical translation, Abellan et al. developed a proof of concept of a 40 cm^2^ large skin graft (Poieskin®) using LIFT. They patterned a human bilayered construct, which includes the dermis that contains fibroblasts and the epidermis that contains keratinocytes. They isolated both types of cells from a 4 cm^2^ biopsy specimen and patterned the fibroblasts on a collagen bed following good manufacturing practices (GMP) and then the keratinocytes on the fibroblast layer. Preclinical testing in mice demonstrated reproducible batches with consistent dimensions and well‐differentiated epidermal layers.^[^ ^120^ ^]^

Despite limitations in the preclinical model and the construct's lack of pigmentation and vascularization, LIFT shows strong potential for treating extensive burns or skin injuries, where donor skin is limited. In comparison with extrusion and inkjet bioprinting, which are susceptible to issues such as shear stress and clogging arising from nozzle utilization or mechanical impairment due to piezoelectric stress, the employment of LIFT facilitates the precise control of cell seeding at the microscale, thereby enabling the creation of high‐resolution patterns while maintaining viability. The present technique has the potential to achieve clinical translation in a realistic manner. It can produce reliable and large‐scale grafts in accordance with GMP practices, as reported by Abellan et al.^[^ ^120^ ^]^ This represents a significant advantage over other techniques that have yet to achieve this level of efficacy, highlighting its ability to achieve precise and reproducible cell deposition at a clinical scale. Consequently, pigmentation could be achieved soon by adding a third cell type and optimizing cell culture conditions. However, further research is required into how to vascularize these grafts for full clinical translation. LIFT technique offered an alternative with the precision and resolution required to create vascularization in organotypic skin models, but also to study conditions like vitiligo, where a precise positioning of the melanocytes within the basal stratum in the epidermis is critical to mimic the tissue and allow the study of the immune reaction involved. This enabled improving nutrient diffusion and supporting cell viability in tissue constructs. Additionally, it brought the opportunity to add immune cell populations relevant for modeling inflammatory skin diseases, where there is an interplay between immunology and the vascular system. Beyond improving the structural complexity, the strategy presented by Douillet et al. is particularly well‐suited for preclinical research into CNISDs, such as hidradenitis suppurativa or acne, where wound healing is compromised. By enabling temporal analysis of the healing process and allowing precise control over the fibroblast: myofibroblast ratio, this model may facilitate the recreation of pathological and immune dynamics, aiding in the optimization of therapeutic interventions for these pathologies.^[^ ^138^ ^]^

### 5.3 Digital Light processing

DLP is a lithography‐based bioprinting technology that uses light to photopolymerize liquid polymeric materials into a solid structure based on a CAD model (Figure 4c). It is based on the deposition of photocurable bioinks layer by layer. One advantage is that it does not require the use of a nozzle, which can compromise cell viability or induce shear stress. It can also operate at high speeds.^[^ ^152^, ^161^ ^]^ This technique brings high viability and accurate organotypic models. Choi et al. developed an FT, vascularized skin model using DLP to evaluate the effects of epidermal growth factor (EGF). The construct remained mechanically stable and viable for up to four weeks, and EGF treatment enhanced the proliferation of both fibroblasts and keratinocytes.^[^ ^152^ ^]^ Similarly, Zhou et al. employed DLP to fabricate a vascularized dermal model, demonstrating the printability and biofunctionality of a GelMA/HA‐NB/LAP‐based bioink. The construct supported neotissue formation and neovascularization upon implantation without eliciting an immune response. Moreover, the study highlighted the influence of printed geometry on cellular behavior; the incorporation of microchannels improved the implantation microenvironment by enhancing nutrient and oxygen diffusion, and moisture exchange (Table 5).^[^ ^139^ ^]^ These complementary strategies demonstrate the versatility of DLP in addressing different limitations of in vitro skin models, whether through the reinforcement of mechanical stability for long‐term culture^[^ ^152^ ^]^ or the optimization of microenvironmental conditions through structural design.^[^ ^139^ ^]^ Furthermore, these approaches showed how the incorporation of vasculature significantly enhances the complexity and physiological relevance of skin models, an aspect that is particularly challenging to achieve through manual fabrication techniques.

### 5.4 Extrusion‐Based Bioprinting

The extrusion‐based bioprinting technique is widely used for bioprinting organotypic skin models with different complexities (Table 5), mainly due to the wide range of possibilities to control printing parameters and the option to work with single or multiple heads, biomaterials, and high cell density bioinks. It´s a versatile and simple technique that possesses a broad spectrum of bioinks that can be used on the same construct, which makes it a cost‐effective technique.^[^ ^14^ ^]^ It uses pneumatic or mechanical pressure to extrude cell‐laden bioinks as filaments from the extrusion nozzle on a printing platform.^[^ ^123^, ^162^ ^]^ To prepare organotypic skin models using this technique, viscous ink is typically mixed with fibroblasts and printed layer by layer to form the dermal compartment. Once this structure is complete, keratinocytes are deposited on top to form the epidermal layer, either manually (e.g, by pipetting) or using a secondary bioprinting method such as inkjet or LIFT, as described earlier.

There are multiple commercial systems for extrusion‐based bioprinting that are user‐friendly and affordable.^[^ ^8^ ^]^ The printing process is carried out based on a CAD model, where the dispensing head is moved along controllable X‐Y‐Z axes (Figure 4d).^[^ ^119^, ^133^ ^]^

Several groups have developed FT models using fibroblasts and keratinocytes and different biomaterials to prepare the bioink (Table 4),^[^ ^95^, ^144^, ^145^, ^146^, ^147^, ^148^ ^]^ all of which demonstrate well‐organized, stratified structures resembling native skin. While the overall architectural outcomes are comparable, the main distinctions among these models lie in the choice of biomaterials and their intended applications, an aspect that will be discussed in the next section about inks.

To further enhance biomimicry, some studies have focused on replicating the basement membrane, which plays a critical role in skin structure and function, and prevents keratinocytes from infiltrating the dermis and forming tumor‐like structures that are not part of native skin. To address this, Derr et al. introduced a cell‐free bioink syringe into their bioprinting setup. This layer was deposited prior to bioprinting the keratinocytes and after bioprint the dermal compartment, enabling the formation of organotypic skin models with improved barrier function,^[^ ^142^ ^]^ whereas Chae et al. advanced the biomimicry of organotypic skin models by attempting to replicate the architectural complexity of rete ridges within the basement membrane. They bioprinted a basement membrane layer, employing a strategically designed lattice‐like filament pattern to recreate the characteristic undulating dermo‐epidermal interface found in native skin.^[^ ^143^ ^]^ Changes in the structure of rete ridges are associated with other biological processes, including aging, characterized by a tendency to flatten, and scar tissue formation, which arises from impaired signaling between fibroblasts and keratinocytes due to incomplete base membrane formation.^[^ ^143^, ^163^ ^]^ These approaches underscore the necessity of discerning the optimal balance between two distinct yet complementary methodologies. On the one hand, there is the provision of the base membrane with straightforward and replicable strategies. On the other hand, there is the emphasis on the architecture and its meticulous replication, capturing the intricacies that are characteristic of physiological processes. The selection of either of these approaches is determined by the specific nature of the research question being addressed.

As already highlighted, the hypodermis is also part of the skin's overall structure and function. Consequently, several studies have focused on incorporating this layer into organotypic skin models. Avelino et al. demonstrated in a bioprinted model that the presence of the hypodermis can modulate the expression of genes involved in key skin processes such as epidermal proliferation and differentiation, thereby enhancing the functionality of in vitro skin constructs.^[^ ^149^ ^]^ Similarly, Moakes et al. bioprinted a trilayered skin model and observed the mobilization of surrounding hypodermal tissue into the construct's hypodermal layer, a process that may improve graft integration.^[^ ^151^ ^]^ Collectively, these works demonstrate a balanced equilibrium between biological fidelity and the complexity of fabrication. The incorporation of the hypodermis enhances functionality from multiple perspectives, though it may consequently increase the likelihood of further standardization in cell culture conditions.

One major limitation of manually fabricated organotypic skin models is the difficulty in replicating complex structures such as vasculature and functional perfusion. Moreover, perfusable vasculature enables immune cell trafficking, which is necessary for the study of inflammation‐driven disease. The absence of vascularized components has long posed a challenge in tissue engineering, as it impairs the recreation of a physiologically relevant tissue microenvironment. In this sense, Liu et al., engineered a vascularized and immunogenic skin model for drug testing in atopic dermatitis (AD). After bioprint the model, they induce the AD phenotype by stimulation with IL‐4 during the ALI culture of organotypic skin models. Their model successfully recapitulated disease‐specific hallmarks and demonstrated enhanced therapeutic efficacy of drug candidates within the vascularized environment.^[^ ^110^ ^]^ In a recent contribution, Peng et al. explore the disease modeling in this case for psoriasis, biofabricating an immunocompetent model that incorporates Th17 cells for psoriasis and drug research, showing the use as a potential preclinical platform to understand pathological mechanisms and drug efficacy assessment.^[^ ^107^ ^]^

In response to increasing restrictions on animal testing in toxicology, Bhar et al. developed a vascularized and immunocompetent model for classifying corrosive and irritant compounds. This platform provided sensitive and reliable assessments, supported by inflammation profiles mediated by incorporated macrophages from the cell line THP‐1 that, prior to being added to the model, were activated to an undifferentiated M0 phenotype, which, in the presence of proinflammatory substances, polarized to M1.^[^ ^96^ ^]^ Another example is provided by Baltazar et al., who constructed a perfusable vascularized skin model, demonstrating that the inclusion of a functional vascular network significantly improves integration into the host tissue upon implantation on immunodeficient mice, compared to the organotypic models without printed vasculature.^[^ ^153^ ^]^

Vascularization has been shown to enhance physiological relevance, thereby facilitating nutrient diffusion, immune cell trafficking, and graft integration, thus enabling a variety of applications in skin modeling. Liu et al. and Peng et al. concentrated on disease modeling and drug testing,^[^ ^107^, ^110^ ^]^ Bhar et al. on toxicology assays,^[^ ^96^ ^]^ and Baltazar et al. on in vivo graft integration.^[^ ^153^ ^]^ While incorporating vasculature is necessary for maintaining functional fidelity, it introduces technical challenges and potential variability compared to non‐vascularized models. This highlights the trade‐off between complexity and reproducibility. Additionally, as is shown in Kim et al ^[^ ^28^ ^]^ FT skin vascularized models allow the study of specific aspects of systemic pathologies like type 2 diabetes. Beyond vascularization, the integration of skin appendages represents another major step toward mimic the complexity of native skin, opening the opportunities to a new generation of tissue and disease modeling.

Recent advances in 3D bioprinting have supported the development of skin models that incorporate elements such as sweat glands, hair follicles, and pigmentation. Zhang et al. developed a 3D bioprinted skin model that integrates sweat glands and hair follicles. To do that, they embedded mesenchymal stem cells (MSCs) with a plantar dermis homogenate (PD) to recreate the sweat gland microenvironment. They embedded the MSC and PD in a gelatin‐alginate ink and bioprinted a cylindrical mesh with a gap between every printed line to form the sweat gland constructs (SG). Later, they isolated fibroblasts and keratinocytes from newborn mice and prepared hair follicle spheroids (HF) that were cultured in hair follicle medium. Once the spheroids were formed, they were seeded on the SG construct, allowed to sediment to the bottom of the SG construct through the gap created previously, and cultured for seven days in HF/SG medium. This platform enabled the study of the reciprocal interactions between sweat glands and hair follicles. The cell heterogeneity within the construct contributed to sustained viability over time, and the co‐culture environment enhanced the in vitro differentiation of both appendage types.^[^ ^154^ ^]^

A different approach was proposed by Motter Catarino et al., who engineered a pigmented vascularized skin model incorporating hair follicles. To do that, they bioprinted the dermal compartment containing fibroblasts embedded in rat‐tail collagen I. Later, they bioprinted the base membrane that was acellular collagen IV ink, following the bioprinting within the previous structure of the hair follicle bioink that contained human follicle dermal papilla cells (DPC) and HUVECs in cell culture medium. Finally, they bioprinted the epidermis that contained keratinocytes and melanocytes. With this platform, Motter Catarino et al showed that the combination of DPC cells and HUVECs can form spheroids inside the dermal compartment, and the keratinocytes and melanocytes on the epidermis migrate, contributing to the formation of the hair follicle, mimicking the hair follicle organization in native skin.^[^ ^155^ ^]^

Another notable example is presented by Jorgensen et al., who bioprinted a skin equivalent designed to mitigate several secondary effects commonly observed post‐grafting, highlighting its potential for improving graft outcomes.^[^ ^156^ ^]^ Although wound healing is a complex and multistep process of tissue reparation, there are inflammatory immune responses that initiatiate and regulate the process; any impairment of this response can trigger to the development of phenomena like fibrosis or chronic immune‐related skin disorders.^[^ ^8^ ^]^ Jorgensen et al. developed a full‐thickness, human‐like skin model incorporating vascularization, pigmentation, and hair follicles. The bioink, composed of fibrinogen, gelatin, glycerol, and hyaluronic acid, was divided into three formulations: a hypodermal bioink with pre‐adipocytes, a dermal bioink containing fibroblasts, follicle dermal papilla cells (FDPCs), and human dermal microvascular endothelial cells (HDMECs), and an epidermal bioink with keratinocytes and melanocytes. These layers were bioprinted sequentially: hypodermis, dermis, and epidermis, then crosslinked with thrombin and cultured for seven days before implantation into full‐thickness wounds in mice or pigs.^[^ ^156^ ^]^ The resulting construct improved wound healing, supported neovascularization, and reduced fibrotic tissue formation. Although melanocytes and follicular cells were included, pigmentation and hair follicle formation were not observed at the graft site in mice, likely due to model‐specific limitations. The authors suggest that preassembling follicular units into spheroids, as shown in prior studies, may be necessary to achieve hair growth.^[^ ^154^, ^155^, ^156^ ^]^

The presented works demonstrate the potential of 3D bioprinting technology in the fabrication of skin appendages with a high degree of similarity to the original tissue. Zhang et al. recreated sweat gland microenvironments, enabling the study of specific cell interactions. Motter Catarino et al. demonstrated pigmented follicle spheroid formation, relevant for the cosmetic industry, and Jorgensen et al. bioprinted vascularized, pigmented, follicular skin equivalents with a clear clinical translation potential.^[^ ^154^, ^155^, ^156^ ^]^ Extrusion‐based 3D bioprinting is particularly suitable to model acne and hidradenitis suppurativa, where the appendages are also involved in the pathology.^[^ ^49^, ^58^ ^]^ A critical component in 3D bioprinting is the ink used to embed the cells. The composition, mechanical properties, and biocompatibility of the biomaterial have an influence on the outcome in skin tissue engineering.

While appendage incorporation enhances structural and functional realism, there is a need to balance between complexity and robustness since current models still face challenges in achieving full maturation and functional integration. Building on these advances in 3D bioprinting can serve as a powerful platform to improve anatomical complexity but also to study the immunological mechanisms in several skin disorders through specific technical strategies to mimic the main hallmarks of each disease, as is shown in section 3. Conditions immune‐driven, such as vitiligo, hidradenitis suppurativa, acne, and chronic spontaneous urticaria, currently lack proper in vitro organotypic models.^[^ ^50^, ^58^, ^67^, ^71^ ^]^ The precision and high resolution that LIFT techniques bring could enable a precise positioning of melanocytes and different immune cell populations in vitiligo and CSU; sacrificial printing could be applied to recreate the sinus tracts in hidradenitis suppurativa, together with extrusion approaches to add different appendages relevant for acne. A comparative summary of the benefits and constraints of the bioprinting techniques explained is shown in **Table** **6**.

**Table 6:** Benefits and constraints of different bioprinting techniques.

3D Bioprinting technique	Benefits	Constraints
*Inkjet*		
– Thermal – Piezoelectric	– Nozzle clogging limited – High cell density – Low manufacturing costs – High speed – Large areas	– Very low viscosity inks – Potential cell sedimentation (need for cell agitation mechanism)
*Extrusion*		
– Pneumatic – Mechanical	– Low cost – High cell density – Versatile – Scalability	– Nozzle clogging – Slow speed – Low resolution – Cell viability impacted by shear stress (ink requirement: shear‐thinning properties)
Laser Induced Forward Transfer (LIFT)	– High resolution – High cell density – High cell viability	– High costs – Limited print volumes – Potential laser irradiation cell sensitivity
Digital Light Processing (DLP)	– High resolution – High speed – High viability	– Viscosity restrictions (limited ink selection)

### 5.5 Inks and their Properties in Skin Bioprinting

In native skin, the ECM provides support but also regulates the polarity of epidermal stem cells, enhances growth, and promotes wound healing.^[^ ^9^ ^]^ The ECM forms a network of fibrous and non‐fibrous constituents, the cells in a tissue. Among the fibrous components, we can find collagen, elastin, and fibronectin fibers, and glycosaminoglycans, proteoglycans correspond to non‐fibrous components, all together determine the mechanical properties of the skin.^[^ ^121^ ^]^ To recreate the architecture of native skin, the biomaterials used to prepare the bioink and bioprint organotypic skin models must mimic the ECM composition^[^ ^131^ ^]^ and tissue functionality, as well as the physical, mechanical, and chemical properties, while also being printable and maintaining the construct integrity after bioprinting.^[^ ^6^, ^127^, ^132^ ^]^

The biomaterials available for bioprinting can be divided into two groups according to their origin: natural and synthetic. One of the challenges in the bioprinting techniques is the lack of equilibrium between those characteristics that allow a material to be highly biocompatible, as is the case with natural biomaterials, and those that provide mechanical properties, printability, and stability over time, as is the case with synthetic biomaterials.^[^ ^132^ ^]^ Because the majority of the organotypic skin models presented in this work have been developed with natural biomaterials, in the next section, we will describe the most commonly used biomaterials in organotypic skin modeling.

### 5.6 Natural Biomaterials

Among naturally derived biomaterials, both whole ECM (decellularized‐dECM)^[^ ^28^, ^137^ ^]^ and its individual components, such as collagen,^[^ ^147^, ^149^, ^150^ ^]^ hyaluronic acid, elastin, laminin, entactin, and dermatan sulfate, are commonly used for preparing organotypic skin models.^[^ ^28^, ^95^, ^119^, ^141^, ^142^, ^143^, ^151^, ^155^, ^156^ ^]^ Other components, such as fibrinogen^[^ ^110^, ^142^ ^]^ and fibrin, platelet‐rich plasma (PRP),^[^ ^96^ ^]^ and gelatin (denatured collagen),^[^ ^95^, ^138^, ^142^, ^146^, ^148^ ^]^ are also used (**Table** **7**). Additionally, other natural polymers like agarose, alginate,^[^ ^144^, ^154^ ^]^ chitosan, pectin,^[^ ^151^ ^]^ nanocellulose,^[^ ^145^ ^]^ and silk fibroin,^[^ ^152^ ^]^ have been used in bioink formulations for bioprinting organotypic skin models.^[^ ^6^, ^123^ ^]^ These materials are generally characterized by having good biocompatibility. However, they often exhibit poor mechanical properties and, in some cases, require extended gelation times.^[^ ^6^ ^]^

**Table 7:** Comparison between natural and synthetic bioinks.

Biomaterials	Characteristics	Examples
Natural	Advantages: – Good biocompatibility – ECM‐related promote cell adhesion, proliferation, and cell migration – Enzymatic degradability Disadvantages: – Lack of proper mechanical and chemical properties – Long gelation times in some cases – Poor printability properties – Contraction of some materials	– ECM derivates: collagen, gelatin, fibrin, hyaluronic acid – Decellularized ECM – Agarose – Alginate – Chitosan – Silk Fibroin – Gelatin methacrylate (GelMA)
Synthetic	Advantages: – Controllable mechanical and chemical properties – Better printability properties Disadvantages: – Lack of biocompatibility – Poor degradability – Lack of adhesion motifs	– Polyethylene glycol (PEG) – Polylactic acid (PLA) – Polycaprolactone (PCL)

Collagen remains the most used biomaterial to prepare organotypic skin models using manual techniques and for the study of skin inflammatory conditions. There are ≈28 types of collagens,^[^ ^121^ ^]^ but the most used are collagen I and, in some cases, collagen IV.^[^ ^155^ ^]^ Collagen I is usually obtained from bovine, rat tail, or porcine sources, but the literature also mentions some works with duck collagen.^[^ ^121^ ^]^ As a key component of ECM, collagen contains multiple cell adhesion domains that support cell proliferation and migration. Although it exhibits low mechanical strength.^[^ ^123^ ^]^ Collagen is generally well‐tolerated by the immune system and does not elicit significant immune rejection or inflammation when grafted.^[^ ^6^ ^]^ The use of collagen also presents some disadvantages for bioprinting, and its use is mainly for inkjet and LIFT bioprinting. One of the disadvantages is the low viscosity, gelling speed, and temperature. Moreover, it possesses poor mechanical properties, and the contractile forces exerted by the fibroblasts contract the entire construct, affecting the dermis volume.^[^ ^8^, ^121^ ^]^

Gelatin is the denatured form of collagen, highly biocompatible due to the RGD adhesion motifs that interact with integrins in the cells, promoting cell adhesion. Has lower antigenicity and cytotoxicity.^[^ ^127^ ^]^ The strength can be handled through concentration and can be thermally crosslinked. Its stability can be improved using a crosslinker like genipin or chemical modification by methacrylation of side chains to produce Gelatin Methacryloyl (GelMA).^[^ ^8^, ^121^ ^]^

dECM from porcine or bovine dermis has been used as a bioink mainly in wound healing applications. The production involves the removal of the cellular component that can have antigens and activate the immune system, preserving the native ECM, which results in a biomaterial non‐immunogenic.^[^ ^8^ ^]^ The use of dECM promotes a better epidermal differentiation compared with organotypic skin models prepared with collagen I.^[^ ^121^ ^]^

Some disadvantages of the most natural materials are the lack of shear‐thinning properties and poor mechanical properties, making it challenging to bioprint using extrusion‐based technologies; for example, they do not gel immediately after printing, driving to a rapid collapse of the filaments and the bioprinted structure.^[^ ^133^ ^]^ Additionally, during the production process, it is very common to find differences in their properties from batch to batch, creating the necessity to adjust the bioprinting parameters every time the batch changes.^[^ ^164^ ^]^

To enhance the mechanical properties of bioinks, several crosslinking strategies has been developed among them^[^ ^165^ ^]^: ionic crosslinking, widely used with alginate where Calcium chloride is added to promote chemical interactions between calcium ions and the alginate; photoiniated through addition of photoinitiators that induce photopolymerization in the biomaterial; and enzymatic where the most used strategy is the interaction between fibrinogen and thrombin to induce fibrin formation. However, each method influences cell behavior and viability; choosing the crosslinking parameters is critical to avoid excessive stiffness that could affect cell viability.^[^ ^166^ ^]^

### 5.7 Synthetic Biomaterials

Synthetic biomaterials are chemically synthesized, meaning that they do not depend on natural sources to be produced^[^ ^123^ ^]^ (Table 7). They show some advantages with respect to natural biomaterials with respect to mechanical properties, resulting in stable structures and low variability batch‐to‐batch. However, these materials often lack adhesion motifs in their chemical structure, which reduces their biocompatibility, presents low biodegradability, and limits their direct application in 3D bioprinting, often requiring additional functionalization steps with natural biomaterials.^[^ ^127^, ^130^ ^]^ Examples of synthetic materials are polyethylene glycol (PEG), polylactic acid (PLA), and polycaprolactone (PCL).^[^ ^6^ ^]^

The biomaterials used to prepare the bioink must be biocompatible,^[^ ^6^, ^132^ ^]^ which means they should be highly porous, to retain high water content, promote cell migration, as well as diffuse nutrients and growth factors from the culture medium efficiently.^[^ ^8^ ^]^ Another important characteristic is that biomaterials should support and promote cell proliferation and differentiation to keep high viability over time, the organotypic models are kept in culture.^[^ ^14^ ^]^ Finally, they should be non‐toxic, and both the material itself and its biodegradation products must not be immunogenic.^[^ ^164^, ^167^ ^]^

The biodegradability of the material is also important to promote tissue remodeling in those applications related to regenerative medicine.^[^ ^167^ ^]^ The interaction of natural and synthetic materials with the immune system will be discussed in the following section.

For a biomaterial to be suitable for 3D bioprinting, it must exhibit favorable rheological properties, among them, appropriate viscosity according to the bioprinting techniques used. Inkjet bioprinting requires the use of low‐viscosity biomaterials, while extrusion‐based bioprinting can manage more viscous biomaterials.^[^ ^157^ ^]^ Additionally, for those biomaterials used in DLP bioprinting, it is important to consider, in addition to the properties mentioned, the optical properties and polymerization mechanism to choose the proper material and bioprinting conditions.^[^ ^164^ ^]^ The shear‐thinning behavior is another property that allows the biomaterial to flow under applied pressure during extrusion and subsequently recover its structure to preserve the geometry of the printed construct. This property facilitates smooth deposition while maintaining shape fidelity. However, excessively high viscosity can increase shear stress on embedded cells, negatively affecting their viability. As such, careful optimization and standardization of bioprinting parameters are essential to ensure the production of structurally stable and highly viable constructs.^[^ ^162^ ^]^

When selecting a biomaterial ink for the development of bioprinted immunocompetent skin models, viscoelasticity should be one important property of the material, as native skin is highly viscoelastic and presents short stress‐relaxing times. Skin models based on collagen hydrogels exhibit similar mechanical properties of the native ECM, such as viscoelasticity. As discussed above, collagen is difficult to bioprint and is mostly used for manual casting. However, such models still lack other key components of the skin ECM, such as elastic fibers, glycoproteins, and proteoglycans. Alginate is commonly used for bioprinting and has been shown to be tunable and exhibit a range of viscoelasticity (with relaxation times τ1/2 from ≈1 h to ≈1 min).^[^ ^168^ ^]^ The fast‐relaxing hydrogels allowed for fibroblast spreading and proliferation, while the slow‐relaxing gels hindered it.^[^ ^168^ ^]^ GelMA is a widely used ink for extrusion‐based bioprinting but exhibits an elastic‐like behavior. It can be rendered viscoelastic by mixing GelMA with alginate^[^ ^169^ ^]^ or with other gelatin species, taking advantage of dynamic bonds.^[^ ^170^, ^171^ ^]^ Other strategies for the molecular design and fabrication of viscoelastic hydrogels have been reviewed in the literature and could be implemented as bioinks for the development of tissue models of CNISDs.

The immune response to biomaterials has been widely studied in medical devices and wound healing research, and some concepts and reactions can be applicable to the development of immunocompetent organotypic skin models. The immune system can be triggered and initiate an immune response in the presence of foreign particles, including natural and synthetic biomaterials, mainly from those of multispecies origin.^[^ ^172^ ^]^ Implanted biomaterials can mediate macrophage polarization to a proinflammatory M1 phenotype. This response is enhanced by hydrophilic surfaces, and biomaterials like alginate and composites of gelatin with PCL, and biomaterials like quitosan activate dendritic and T cell responses. Additionally, fibroblast has paracrine effects in switching the macrophage response to a fibrotic condition.^[^ ^173^ ^]^ This information can be relevant in the production of immunocompetent skin models to study inflammatory conditions, since a basal immune activation from the biomaterial can lead to wrong conclusions, mainly in those skin pathologies where the immune response is not completely clear or characterized.

## 6 Challenges and Opportunities in 3D Bioprinting of Immunocompetent Skin Models

This review shows several strategies to vascularize models that can be suitable to capture microvascular involvement in diabetes and CSU. The possibility of controlling the cell deposition and materials properties could open the opportunity to a new generation of organotypic skin models, providing a disease‐driven rationale for selecting a given technique, beyond enhancing structural fidelity. Given the limited number of existing immunocompetent skin models generated through 3D bioprinting, this represents a significant opportunity to expand the field and harness the full potential of the technology for dermatological research and therapeutic development. Even in well‐studied conditions like psoriasis and atopic dermatitis, developing bioprinting strategies could improve drug testing in more physiological immunocompetent and relevant models.

As demonstrated in Tables 1 and 4, a discrepancy emerges between integrating immune components into organotypic skin models and the fabrication strategies employed. Over the past fifty years, most skin models and substitutes have relied on manual techniques.^[^ ^114^, ^174^, ^175^ ^]^ This is not an exception for immunogenic and immunocompetent skin models. These approaches provide a fundamental structural framework for exploring biological pathways, characterizing immune cell responses, and determining cytokine profiles. Conversely, the field of 3D bioprinting research has been developing for nearly two decades^[^ ^78^, ^175^ ^]^ with a focus on addressing the architectural and anatomical complexity of by adding skin appendages, replicating rete ridges, and vascularization. Moreover, the development of biomaterials is crucial for creating inks that exhibit properties analogous to mechanical and biological tissue properties.

**Figure 5:**
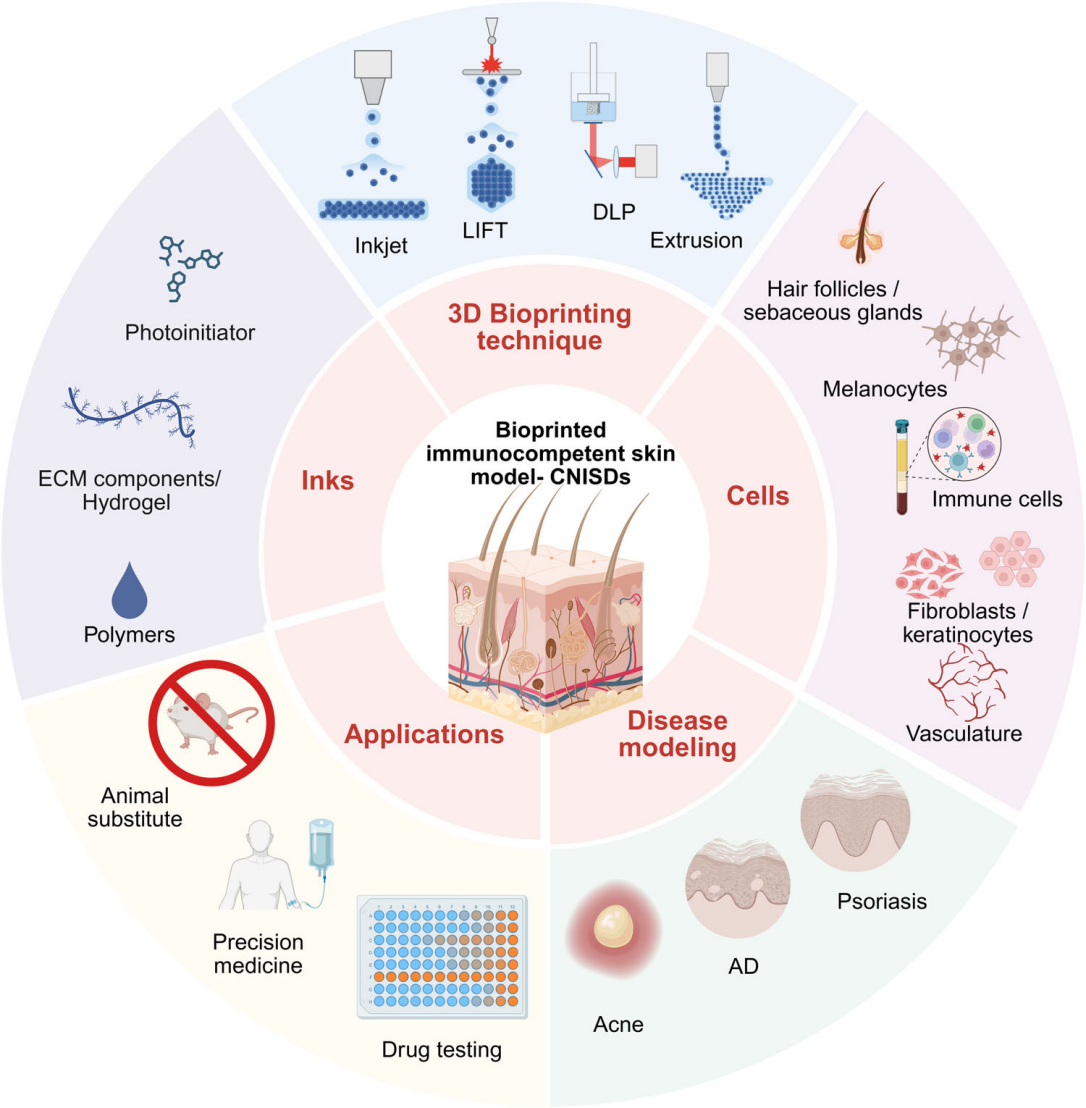
Summary of the requirements to develop 3D bioprinted immunocompetent skin models for CNISDs. Created in BioRender. Ulloa, A. (2025) https://BioRender.com/pmbv6y6.

The contrast between manual and bioprinted models reflects their historical trajectories: manual models have been optimized to bring biological relevance first, while 3D bioprinting has been working on overcoming engineering barriers and bringing physiological relevance through mimicking the complex structures that compose the skin before addressing immune complexity. In recent years, 3D bioprinting technologies have been used, as previously described, to create models that resemble common CNISDs.^[^ ^28^, ^95^, ^96^, ^107^, ^110^ ^]^ However, incorporating immune cells into these models poses additional challenges, including sourcing these cells and addressing allogenic immune responses. It is critical to address these challenges to advance beyond the development of disease models toward the establishment of fully immunocompetent skin models for CNISDs (**Figure** **5**).

The use of 3D bioprinting technologies has progressed in improving the anatomical complexity of organotypic skin models. While challenges still remain, some of them can perhaps be solved with 3D bioprinting; others are beyond bioprinting. In any case, it also represents an opportunity to continue research, looking for an ideal skin model.

### 6.1 Challenges that 3D Bioprinting can Tackle

#### 6.1.1 Addition of Skin Appendages

Advances in 3D bioprinting have enabled the development of organotypic skin models that include appendages such as hair follicles, sweat glands, and pigment (melanocytes) for different purposes,^[^ ^154^, ^155^, ^156^ ^]^ representing a substantial step forward in tissue engineering. Such developments are not feasible with manually cast models, while high‐resolution 3D bioprinting technologies bring the opportunity to control the positioning of skin appendages in complex organotypic skin culture.^[^ ^16^, ^176^ ^]^

#### 6.1.2 Addition of Vascularization and Fluid Flow

One of the main current limitations in the development of organotypic tissue models in vitro is the lack of vascularization, which impacts the diffusion of nutrients from the cell culture medium to maintain viability, but also to study immune dynamics in different scenarios.^[^ ^177^, ^178^ ^]^ Studies using 3D bioprinting approaches have demonstrated the impact of the presence of a perfusable vasculature in the maturation and function of skin equivalents,^[^ ^28^ ^]^ and their response to treatments.^[^ ^110^ ^]^ Indeed, 3D bioprinting brings the technology necessary to incorporate the vascular component and thus make dynamic models – in contrast to static models which have mostly been developed to date –, which could be coupled with organ‐on‐chip technologies for hybrid biofabrication approaches, leading to perfusable models with controlled fluid flow, by means of pumping^[^ ^179^ ^]^ or pump‐free methods employing gravity‐driven flow.^[^ ^180^ ^]^

#### 6.1.3 Addition of Skin Immune Components

Most immunogenic and immunocompetent models found in the literature have been developed using manual techniques. There are efforts to use 3D bioprinting for the study of inflammatory skin diseases like AD^[^ ^110^ ^]^ and diabetes,^[^ ^28^ ^]^ showing the models are robust enough for drug testing, even as a model to classify skin irritants, and finally substitute the use of animals.^[^ ^96^ ^]^ In immunological research, the vasculature plays an important role in the immune response, and together with the vasculature, it is possible to mimic the dynamic of native skin between the vasculature and the immune system, in complex diseases like vitiligo, hidradenitis suppurativa, chronic spontaneous urticaria, and acne.

#### 6.1.4 Impact of Bioink and Bioprinting Parameters on Immune Functionality

Together with point 2, there is a lack of standardized protocols to evaluate immune function post‐bioprinting. Variables like printing mechanics, bioink rheological properties, and material chemistry can affect the cell behavior in the material and, in some cases, cause cell death and influence the cell phenotype, due to shear stress or toxicity.^[^ ^165^, ^181^ ^]^ These parameters should be carefully optimized to not affect the viability and the phenotype or cell differentiation in the models.^[^ ^165^ ^]^ Clear benchmarks and workflows need to be established in the pursuit of immunocompetent skin models for high‐throughput applications. In this regard, Martin‐Saldaña et al., from an interdisciplinary point of view, summarize the strategies available to characterize ECM hydrogels, including 3D bioprinting.^[^ ^182^ ^]^

#### 6.1.5 Improving Biomaterial Properties and Hardware

Most of the models presented in this work were established using natural biomaterials,^[^ ^28^, ^30^, ^40^, ^74^, ^89^, ^90^, ^91^, ^92^, ^95^, ^96^, ^97^, ^98^, ^99^, ^100^, ^101^, ^102^, ^103^, ^105^, ^106^, ^108^, ^110^, ^137^, ^140^, ^142^, ^143^, ^144^, ^145^, ^146^, ^147^, ^149^, ^150^, ^151^, ^154^, ^155^, ^156^, ^161^ ^]^ and during the production of the inks, there is batch‐to‐batch variability,^[^ ^182^ ^]^ which raises the need for constant standardization of bioprinting parameters and biocompatibility evaluation. There is a poor reproducibility of matrix sources, which makes it difficult to obtain reproducible models. The poor mechanical properties of natural biomaterials^[^ ^133^ ^]^ have also improved the development of new conditions and accessories in the printers to preserve the bioprinted shape before crosslinking the construct.

### 6.2 Challenges beyond 3D Bioprinting

#### 6.2.1 Cell Source

Given the immune system's sensitivity to non‐self‐antigens, selecting appropriate cell sources remains a challenge. Most of the immunocompetent skin models use cells obtained from buffy coats or isolate and polarize PBMCs, which do not fully reflect the phenotype and function of tissue‐resident immune cells.^[^ ^94^ ^]^ Additionally, location‐specific and systemic factors can influence the behavior and outcomes of the developed model. An ideal in vitro immunocompetent skin model should use autologous cells to minimize allogenic immune responses unrelated to the pathology being studied. Additionally, protocols for isolating and differentiating specific immune cell populations require further optimization to enhance yield, reproducibility, and efficiency. These processes are often labor‐intensive and demand substantial amounts of reagents, antibodies, and interleukins to achieve targeted immune polarization. Moreover, significant variability exists due to donor‐dependent factors, the limited lifespan of primary samples, versus the densities required for 3D bioprinting,^[^ ^165^ ^]^ and other biological variables, further complicating standardization efforts. Progress in iPSCs technology and bioreactor‐based cell expansion systems offers possibilities that could help to tackle this challenge and bring the opportunity to develop autologous, multilineage skin cells.^[^ ^12^, ^165^ ^]^

#### 6.2.2 Multicellular Organotypic Cell Culture Media

Nutritional requirements of multicellular skin organotypic cultures may antagonize or inhibit the proper development, differentiation, or functionalization of other components within the same model, resulting in a reduced lifespan of these complex structures.^[^ ^30^, ^156^ ^]^ Co‐culture conditions are a critical feature to consider in the development of immunocompetent skin models. Griffoni et al. determine that the culture of organotypic skin models in macrophage cell culture medium impairs epidermal differentiation, but if this is changed to skin differentiation media, the macrophages express more of an M2 phenotype, which inhibits inflammation. Cell culture media should provide the components to ensure proper growth of the different cell types in a model and allow a proper interplay between all the cell types that are in the model.^[^ ^30^ ^]^

#### 6.2.3 Interplay with the Microbiome

Integrating elements of the skin microbiome could further strengthen the physiological relevance of these models, especially as emerging evidence links microbiome imbalances to certain skin disorders, as previously described in the context of acne.^[^ ^22^, ^50^ ^]^ Despite growing interest in mimicking immune responses in organotypic skin models for inflammatory diseases, a major limitation remains largely unexplored: the inability to replicate the chronic nature of these conditions. As examples, clinical evidence shows that diseases like psoriasis and vitiligo involve distinct subtypes, persistence, and patient variability.^[^ ^183^, ^184^ ^]^ Similarly, hidradenitis suppurativa features recurrent cycles of inflammation, rupture, and healing, leading to long‐term tissue remodeling and hypertrophic scarring.^[^ ^1^, ^57^, ^58^ ^]^ Most *in vitro* models discussed in this work have limited culture duration, failing to capture prolonged immune activity, remodeling, and relapsing patterns typical of chronic inflammation. This limits their relevance for studying disease progression and long‐term therapeutic effects. Developing strategies to extend the model´s lifespan in culture is a necessary next step toward better reflecting in vivo pathophysiology.

## 7 Conclusion

The use of manual techniques allowed the development of highly relevant immunocompetent organotypic skin models for several skin inflammatory conditions, but the use of 3D bioprinting can help to scale them up and further advance immunological and disease modeling research. While hurdles concerning cell variability, biomaterial choice for bioinks, and cell culture media that support all cell types persist, addressing them will be an opportunity for the successful integration of immune components and enhancing the functionality of organotypic skin models. Advances in 3D bioprinting technologies can revolutionize the fabrication of such models and enable us to study and better understand immune‐related skin diseases.

The next decade should move beyond incremental advances toward developing fully integrated, immunocompetent skin models that incorporate vascularization, appendages, immune components, and microbiome interactions into long‐lived, standardized platforms for CNISDs studies. A critical milestone to achieve is the extension of the cell or tissue culture duration and regenerative capacity to model the chronic and relapsing nature of CNISDs. Finally, the successful overcoming of these challenges could result in the establishment of robust platforms for drug testing, translational applications, and ultimately the replacement of animal models, facilitating the development of patient‐specific systems to apply in precision medicine.

## Conflict of Interest

The authors declare no conflict of interest.
